# The Life History of Learning Subsistence Skills among Hadza and BaYaka Foragers from Tanzania and the Republic of Congo

**DOI:** 10.1007/s12110-021-09386-9

**Published:** 2021-05-13

**Authors:** Sheina Lew-Levy, Erik J. Ringen, Alyssa N. Crittenden, Ibrahim A. Mabulla, Tanya Broesch, Michelle A. Kline

**Affiliations:** 1 Department of Psychology, Simon Fraser University, Burnaby, B.C. Canada; 2 Department of Archaeology and Heritage Studies, Aarhus University, Aarhus, Denmark; 3 Department of Anthropology, Emory University, Atlanta, GA USA; 4 Department of Anthropology, University of Nevada, Las Vegas, Las Vegas, NV USA; 5 Department of Archaeology and Heritage, University of Dar es Salaam, Dar es Salaam, Tanzania; 6 Department of Life Sciences, Centre for Culture and Evolution, Brunel University London, Uxbridge, UK

**Keywords:** Social learning, Foragers, Life history, Cultural transmission, Subsistence skills

## Abstract

Aspects of human life history and cognition, such as our long childhoods and extensive use of teaching, theoretically evolved to facilitate the acquisition of complex tasks. The present paper empirically examines the relationship between subsistence task difficulty and age of acquisition, rates of teaching, and rates of oblique transmission among Hadza and BaYaka foragers from Tanzania and the Republic of Congo. We further examine cross-cultural variation in how and from whom learning occurred. Learning patterns and community perceptions of task difficulty were assessed through interviews. We found no relationship between task difficulty, age of acquisition, and oblique transmission, and a weak but positive relationship between task difficulty and rates of teaching. While same-sex transmission was normative in both societies, tasks ranked as more difficult were more likely to be transmitted by men among the BaYaka, but not among the Hadza, potentially reflecting cross-cultural differences in the sexual division of subsistence and teaching labor. Further, the BaYaka were more likely to report learning via teaching, and less likely to report learning via observation, than the Hadza, possibly owing to differences in socialization practices.

**Supplementary Information:**

The online version contains supplementary material available at 10.1007/s12110-021-09386-9.

## Résumé

Certains aspects de l'histoire de la vie humaine et de la cognition, comme la longue enfance et le recours intensif à l'enseignement, ont théoriquement évolué pour faciliter l'acquisition de tâches complexes. Le présent article examine empiriquement la relation entre la difficulté des tâches de subsistance et l'âge d'acquisition, les taux d'enseignement et les taux de transmission oblique chez les chasseurs-cueilleurs Hadza et BaYaka de Tanzanie et de la République du Congo. Nous avons également examiné les variations interculturelles sur la façon dont l'apprentissage se fait et auprès de qui. Les modèles d'apprentissage et les perceptions de la communauté concernant la difficulté des tâches ont été évalués par le biais d'entretiens. Nous n'avons trouvé aucune relation entre la difficulté de la tâche, l'âge d'acquisition et la transmission oblique, et une relation faible mais positive entre la difficulté de la tâche et les taux d'enseignement. Alors que la transmission entre personnes de même sexe était normative dans les deux sociétés, les tâches classées comme plus difficiles étaient plus susceptibles d'être transmises par les hommes chez les BaYaka, mais pas chez les Hadza, ce qui reflète potentiellement les différences interculturelles dans la division sexuelle touchant le travail impliqué dans la subsistance et l'enseignement. En outre, les BaYaka étaient plus susceptibles que les Hadza de déclarer qu'ils apprenaient au moyen de l'enseignement et moins susceptibles d'apprendre par observation, peut-être en raison de différences dans les pratiques de socialisation.

Humans rely on a vast amount of cultural knowledge for survival in the diverse environments we inhabit (Boyd and Richerson 1985). This cultural knowledge is improved upon and transmitted from one generation to the next, leading to the development of highly specialized and often complex subsistence knowledge, technology, and skill (Boyd et al. 2011; Hewlett and Cavalli-Sforza 1986). Unique features of human life history and cognition have been theoretically tied to such task complexity. For example, human childhood may have evolved as an extended period for learning to extract the difficult-to-acquire resources inherent to our foraging niche (Kaplan et al. 2000). Because it is specialized and widespread, human teaching may also have evolved to facilitate the transmission of complex knowledge (Csibra and Gergely 2011).

However, to our knowledge, only two studies have empirically examined the relationship between task difficulty and how, when, and from whom learning occurs. Kline et al. (2013), working with rural Fijians, found that task difficulty was positively related to later age of acquisition and learning from adults other than parents. Working with the Tsimane in Bolivia, Schniter et al. (2015) found that whereas most basic tasks are learned by adolescence, tasks considered more difficult to perform are learned in mid to late adulthood. Building on these works, the present paper investigated social learning in two forager societies: the Hadza of Tanzania and the BaYaka of the Republic of Congo. Our interviews focused on learning subsistence tasks because these occur daily, vary in difficulty, and because foraging characterized our species’ defining subsistence strategy during 95% of our evolutionary history (Marlowe 2005). We specifically examine the relationship between subsistence task difficulty and age of acquisition, transmission method, and transmission pathway. Further, we examine whether tacit and explicit knowledge developed simultaneously, as well as whether knowledge transmission primarily occurred between same-sex individuals. We compare two forager societies in order to better understand how subsistence strategies and cultural practices shape learning. In what follows, we outline the rationale and hypotheses for the present study.

## Background

Humans have longer childhoods, extended parental provisioning, and larger brains than other members of our clade (Bogin 1997; Kaplan et al. 2000; Lancaster et al. 2000; Robson and Wood 2008; but see Miller et al. 2019). Although these features may be the result of selection for longer human life spans generally (Charnov and Berrigan 1993), the Embodied Capital Hypothesis instead posits that human life history features are linked to the extensive learning required to successfully perform the subsistence activities inherent to our skill-intensive foraging niche, such as hunting, plant collecting, and plant processing (Kaplan et al. 2000). Long learning periods for high-skill tasks may be attributed to a variety of factors. First, children are usually not net producers, likely because young children often lack the physical or mental abilities needed to perform complex tasks (Bock 2002; Demps et al. 2012). For example, although practice is necessary for the successful processing of *mongongo* nuts, children in the Okavango Delta nonetheless require baseline strength and coordination before they can start to practice (Bock 2002). Second, many tasks are modular and cumulative, such that aspects of a task must sometimes be learned sequentially (Demps et al. 2012; Ellen 2009). For example, before learning to cut honeycomb from trees, individuals must first learn to climb (Demps et al. 2012). Finally, many especially-high-skill tasks require extensive experience for success. Among the Tsimane, Gidra, and Ache, for example, hunting skill peaks in mid to late adulthood and is strongly correlated with experience, independent of strength and size (Gurven et al. 2006; Ohtsuka 1989; Walker et al. 2002).

That said, not all resources exploited by humans require cumulative experience for success. Meriam and Mardu children are efficient collectors of marine resources and small prey, and size rather than skill seems to constrain their foraging returns (Bird and Bliege Bird 2002, 2005; Bliege Bird and Bird 2002, see also Blurton Jones et al. 1994; Crittenden et al. 2013; Hawkes et al. 1995; Tucker and Young 2005). Thus, children may be proficient foragers from an early age, even though high-skill tasks are learned later. For example, among the Tsimane, most food production, craft production, and childrearing skills are acquired by adolescence, but proficiency at high-skill tasks, such as making boats or ceramic vessels, peaks in mid to late adulthood (Schniter et al. 2015). Similarly, Fijians begin to learn easier tasks in terms of skill (but not strength) in middle childhood, whereas learning more skilled tasks starts in adolescence (Kline et al. 2013).

Beyond differences in when learning occurs, humans learn in a variety of ways, and each learning strategy has associated costs and benefits. Individual learning leads to the development of innovations that are adapted to the local ecology but can be costly because many trials are needed before a useful innovation is developed (Borenstein et al. 2008). In addition to learning individually, humans also acquire knowledge socially, such as through observation and teaching. Observational learning involves observing another individual’s behavior and copying their actions or goals immediately or at a later time (Heyes 2011). Teaching can be broadly defined as “a behavior evolved to facilitate learning in others” (Kline 2015:2), and allows for the accurate transmission of complex knowledge through subtle cues such as gazing and pointing, and through our capacity for instruction through language (Castro and Toro 2014; Csibra and Gergely 2011; Fogarty et al. 2011; Tomasello 1999). Observational learning is less costly than teaching in the sense that it requires effort only from the learner. However, learners may copy suboptimal aspects of observed behavior, or they may make copying mistakes without additional guidance (Castro and Toro 2014). Although teaching is more energetically costly than observational learning because both the learner and teacher must commit time and energy to knowledge transfer (Csibra and Gergely 2006; Kline 2015), teachers call attention to relevant stimuli, and as a result, learners are less likely to attend to irrelevant aspects of a behavior (Castro and Toro 2014; Kline 2016). Thus, teaching may have evolved to facilitate the transmission of opaque or complex cultural artifacts and instrumental knowledge (Csibra and Gergely 2006, 2011; but see Caldwell et al. 2018). However, to our knowledge, only one study has empirically investigated this claim. Kline et al. (2013) found no association between high-skill tasks and teaching among Fijians. More research is needed to investigate whether the theoretical association between teaching and task difficulty is supported.

Whom individuals learn from may also be related to task complexity. Vertical transmission—or transmission which occurs from grandparent/parent to child—is highly conservative as it leads to low rates of change from one generation to the next and maintains interhousehold variation within communities (Cavalli-Sforza et al. 1982; Hewlett and Cavalli-Sforza 1986). Oblique transmission occurs from individuals of the parental generation (other than parents) to members of the offspring generation. If knowledge is obtained by many from a small number of individuals, oblique transmission can lead to high uniformity of knowledge between members of the same generation. Lastly, horizontal transmission involves two or more people from the same age cohort sharing information (Reyes-García et al. 2009). Horizontal transmission can lead to the adoption of innovations within a generation as knowledge quickly passes from one individual to the next (Hewlett and Cavalli-Sforza 1986).

Henrich et al. (2008) and Reyes-García et al. (2016) proposed a multistage model for understanding how learning pathways change across the life course according to individual skill and access to models. In infancy and early childhood, individuals primarily learn vertically from parents because parents are easily accessible, and because transmitting adaptive information to their children improves parents’ inclusive fitness (Eyssartier et al. 2008; McElreath and Strimling 2008; Reyes-García et al. 2016). In middle childhood, much of children’s time is spent in child-only groups, playing and working, and children likely learn a majority of the basic subsistence tasks needed in adulthood horizontally during this time (Boyette and Hewlett 2017; Lew-Levy, Kissler et al. 2020; Maynard and Tovote 2009; Quinlan et al. 2016; Salali et al. 2016, 2019; Zarger 2002). By adolescence and into adulthood, when most basic foraging competencies have been acquired, learners may seek to acquire more complex knowledge obliquely from experts outside the family or the friend group (Aunger 2000; Dira and Hewlett 2016; Garfield et al. 2016; Hewlett 2013, 2016, 2021; Reyes-García et al. 2009). In sum, low-skill tasks performed by most or all of the population are acquired earlier in life from easily accessible models, such as parents and peers. Because high-skill tasks may be more specialized, they are more likely learned from specific models who have expertise, and thus, oblique transmission is potentially more common for these domains.

While skill difficulty may influence learning, other factors may also determine from whom individuals learn. Because all small-scale societies maintain a sexual division of labor (Brown 1970), Hewlett and Cavalli-Sforza (1986) proposed a sexual division of teaching labor*,* in which sex-specific tasks are taught within same-sex dyads (e.g., men teaching hunting to boys, women teaching cooking to girls). Beyond teaching, children are also more likely to identify with, observe, and imitate same-sex models (Bock and Johnson 2004; Draper 1975; Froehle et al. 2019; Gosso et al. 2007; Lew-Levy and Boyette 2018; Salali et al. 2019). Several studies note that same-sex transmission is central to learning hunting, honey collecting, foraging, plant processing, and craft production (Boyette and Hewlett 2017; Demps et al. 2012; Flannery 1953; Hagen et al. 2016; Hewlett 2012; Hewlett and Cavalli-Sforza 1986; Lew-Levy et al. 2017). Thus, same-sex transmission likely facilitates the acquisition of sex-specific tasks.

Finally, while this paper is primarily concerned with tacit knowledge—or implicit “know how” (Nonaka 1997; Ryle 1946)—tacit and explicit knowledge are theoretically related because individuals may only develop explicit knowledge as they gain experience in the task at hand. In other words, tacit and explicit knowledge may develop simultaneously (Gallois et al. 2015; Koster et al. 2016). Explicit knowledge can also be gained without firsthand experience—for example, through stories (Scalise Sugiyama 2017; Weissner 2014). In the few studies that empirically examine the relationship between these two knowledge forms, results have been mixed. Knightley et al. (2013) examined the relationship between the types of wood and feathers used for arrow shafts and fletching and the peer evaluations of arrow quality made by Tsimane participants. They also examined the relationship between plants used for dyes and peer evaluations of bags produced by the participants. The authors found no strong association between explicit and tacit knowledge, potentially because measures of explicit knowledge do not always capture an individual’s ability to apply this knowledge. On the other hand, Koster et al. (2016) found that Mayangna and Miskito ability to identify fish species was highly correlated with fishing ability, whereas true/false knowledge about fish behavior and fishing technique was not. Research on honey collecting among the Jenu Kuruba further suggests that explicit knowledge about bee ecology and medicinal uses for honey emerged simultaneously with tacit knowledge related to honey collecting (Demps et al. 2012).

## The Present Study

Taken together, the research reviewed above outlines the theoretical association between task difficulty, skill ontogeny, teaching, and learning pathways. However, empirical support for these theoretical relationships is limited for several reasons. First, as noted, few studies on the topic exist. To our knowledge, only two studies directly examined the relationship between task difficulty and age of acquisition (Kline et al. 2013; Schniter et al. 2015), and only Kline et al. (2013) directly investigated the relationship between learning and task difficulty. Furthermore, the expected positive relationship between task complexity and rates of teaching has not been supported with Fijian data (Kline et al. 2013). Second, no comparative studies employing the same methodology in two or more settings have been conducted, making it difficult to examine cross-cultural variation in learning simple vs. difficult tasks (Kline et al. 2018).

To fill these gaps, the present paper examines how subsistence task difficulty influences how, when, and from whom learning occurs using interview data from Hadza and BaYaka foragers. Following Kline et al. (2013), we hypothesize that (H1) tasks ranked as more difficult are learned at a later age than tasks ranked as less difficult (see also Schniter et al. 2015); (H2) tasks ranked as more difficult are more likely to be learned via teaching in comparison with tasks ranked as less difficult; and (H3) tasks ranked as more difficult are more likely to be learned obliquely than tasks ranked as less difficult. We further hypothesize that (H4) same-sex transmission is more likely than opposite-sex transmission and (H5) tacit and explicit knowledge are positively correlated.

By comparing two forager populations, the present study considers how similarities and differences in subsistence and cultural practices lead to variation in social learning. Like other subtropical foragers, both the Hadza and BaYaka share the cultural values of egalitarianism, sharing, and personal autonomy (Gardner 1991; Hewlett et al. 2011; Lavi and Friesem 2019; Peterson 1993; Woodburn 1982). These cultural values result in limited gender and age hierarchy, a lack of formal leadership, and strong taboos against coercing others. Nonetheless, the Hadza and BaYaka differ in subsistence practices, sexual division of labor, and child socialization. In what follows, we describe the ethnographic settings for our research, paying special attention to these potential sources of cross-cultural variation in learning.

## Study Sites

The BaYaka inhabit the tropical rainforest of the Congo Basin (Lewis 2002).^1^ The BaYaka subsist on diverse forest resources, including fish, hunted meat, insects, tubers, liana fruit, nuts, mushrooms, and greens. Honey collected from stinging and stingless bees is an important resource during the dry season (Bahuchet 1988). The BaYaka also maintain small horticultural gardens where they grow plantains, cassava, and maize. The BaYaka surveyed for the present study live in the northern region of Congo-Brazzaville, where approximately six months of the year are spent in a village setting, though overnight fishing, hunting, and gardening trips are common during this time. Approximately two months of the year are spent in forest camps during caterpillar season, and approximately four months are spent participating in dam fishing in forest ponds. Because the community surveyed here is accessible by boat or foot only, market integration is minimal. BaYaka participate in garden labor and hunting with firearms for their Bondongo farmer neighbors, for which they may receive market goods, such as flashlights, machetes, and radios (Boyette et al. 2019; Grinker 1994; Joiris 2003; Rupp 2014). Two schools were functioning in the village at the time of data collection; a public school which served both BaYaka and Bondongo children and a BaYaka-specific school aimed at preparing children for entry into the public school system. However, school attendance by BaYaka children is sporadic, with children frequently missing months of school during the fishing season (Kamei 2001). Few adults, and especially women, reported ever attending school. Even among children who frequently attended school, literacy rates were low.

The Hadza are seminomadic mixed-subsistence foragers who live in the arid savanna woodlands of northern Tanzania (Hawkes et al. 2001; Marlowe 2010). The Hadza number approximately 1000 individuals, a majority of whom reside in permanent settlements where they participate in foraging and wage labor and rely on a predominantly agricultural diet (Marlowe 2010). Only about 150 Hadza currently live in bush camps. Whereas Hadza camps historically moved every two to three months (Marlowe 2010), many camps are now semi-permanent (Crittenden and Blurton Jones 2019). Hunted and gathered resources, including game meat, honey, tubers, berries, and baobab fruit, are dietary staples in bush camps (Marlowe and Berbesque 2009). Maize and other domesticated grains, donated by missionaries and/or aid organizations, provided as payment by ethnotour companies and/or researchers, acquired by trade, or purchased from neighboring pastoralists are increasingly available in bush camps (Crittenden et al. 2017; Gibbons 2018; Yatsuka 2015). Despite the recent integration of domesticated grains into a historically wild-food-based diet, children continue participate in food collection (Pollom et al. 2020). Bush-dwelling Hadza have access to cell phones, small solar panels, bicycles, and market goods such as cooking pots, beads, knives, nails, blankets, and clothing (Crittenden 2019). These goods are purchased by Hadza themselves or are gifts from researchers and aid workers. A recent study found that 82% of bush-dwelling Hadza children reported having attended school (Pollom et al. 2020). Data for the present study were collected in bush camps when children were not attending school.

Although both the Hadza and BaYaka maintain a sexual division of labor, this division is more pronounced among the Hadza (Marlowe 2007). Hadza men are responsible for hunting small and large game using bows and poison-tipped arrows, and for collecting honey from stinging and stingless bees. Women never hunt, instead focusing their food collection efforts on plant foods, including tubers, baobab, and berries. While BaYaka men do a majority of the hunting, men often encourage women to participate in some aspects of hunting, such as checking traps. Net hunting, which is less common today than in the recent past, also involves the efforts of both men and women for success (Noss and Hewlett 2001). BaYaka women are the primary collectors of tubers, mushrooms, caterpillars, and other forest products, though men frequently participate in these activities as well (Hewlett 1991; Marlowe 2007). These cross-cultural differences in the sexual division of labor are apparent in childhood; by adolescence, there is minimal overlap between the subsistence activities of Hadza males and females (Froehle et al. 2019), while few sex differences in time allocation to subsistence work have been found among BaYaka children and adolescents (Ellis-Davies et al. in press; Lew-Levy et al. 2019). The present paper considers whether sex differences in knowledge transmission reflect the sexual division of labor of each society.

Our previous research suggests that both Hadza and BaYaka children learn through participation in play and work activities, which primarily occur in the multi-aged, mixed-sex playgroup, outside the purview of adults (Lew-Levy et al. 2019, 2020a). In these playgroups, children often correct, assign, and demonstrate how to perform subsistence tasks, leading to high rates of child-to-child teaching. However, BaYaka children received more adult teaching as they aged, consistent with the multistage learning model (Reyes-García et al. 2016). This same pattern was not found among the Hadza. Differing ethnotheories regarding the role of adults in knowledge transmission may explain this finding. Among the Hadza, adults do not view themselves as the primary teachers of children. Rather, adults emphasize learning through participation in subsistence activities (Crittenden 2016a). As a result, Hadza children are active foragers, regularly producing 25–50% of their daily caloric needs (Crittenden et al. 2013). Hadza parents facilitate children’s participation in foraging by making small functional bows and digging sticks. Hadza children are encouraged to use these tools while foraging with peers (Crittenden 2016b). Among the BaYaka, adults report teaching as an important parental responsibility (Boyette et al. 2019), and BaYaka families often forage together (Lew-Levy et al. 2019). Although BaYaka parents also make children small versions of tools, they are less likely to be functional, and children are rarely encouraged to use these tools during subsistence activities. Further, while BaYaka children are enthusiastic foragers, their foraging returns are minimal (Hagino and Yamauchi 2016). In sum, whereas BaYaka children may receive more direct forms of teaching from adults as they grow older, Hadza parents may provide children with more opportunities to participate in subsistence activities from an early age, after which learning using other mechanisms, such as observation, becomes more common. The present paper considers whether variation in socialization practices influences when knowledge is acquired, and how.

## Methods

### Data Collection

SLL collected data for the present project among the BaYaka in June through September 2016 and 2017, and among the Hadza in March and April 2017 with the help of IAM.

#### Preliminary Interviews

Preliminary interviews were conducted with 20 Hadza (*N*_female_ = 10) and 20 BaYaka (*N*_female_ = 10) adults. Participants were asked to free-list the subsistence tasks they considered important in their community. Hadza participants listed five subsistence domains (Table 1). BaYaka participants listed 17 subsistence tasks (see Table S1 in the ESM for raw free-list responses) which we categorized into larger subsistence domains (e.g., net hunting → Hunting and trapping; Table 1). For each of the nominated subsistence domains, participants were also asked to describe how the task is usually undertaken.

**Table 1:** Subsistence domains, number of participants, and number of female participants who listed each domain

Subsistence Domains	No. of Nominations	No. of Female Nominations
*Hadza*		
Honey	20	10
Hunting	20	10
Tubers	17	9
Baobab	10	4
Berries	7	4
*BaYaka*		
Tubers	15	9
Hunting and trapping	12	5
General collecting	10	6
Honey	7	3
Garden	6	4
Fishing	5	0
Basketry	4	4
House building	2	0
Cooking	1	1
Bowl making	1	0

#### The Questionnaire

Using task descriptions, participant observation, and the expertise of local informants and interpreters, SLL generated a list of pieces of tacit knowledge needed to successfully complete each task for each society. Because house building and bowl making were infrequently mentioned, and not directly related to subsistence, we did not develop questions related to these activities in the BaYaka questionnaire. Further, although the Hadza did not mention cooking as an important subsistence task, cooking is nonetheless necessary to preparing many foods for consumption, and thus, questions related to this task were developed in the Hadza questionnaire.

In some cases, different subsistence domains included some similar underlying knowledge. For example, the ability to weave baskets for carrying foraged foods, for collecting honey, and for fishing relies on BaYaka participants’ knowledge of weaving. However, each of these basket types differs in material selection, weaving patterns, and contextual ecological knowledge. Further, men and women both participate in carrying and honey basket weaving, whereas men are the primary weavers of fish baskets. In such cases, a separate question for each task was developed to adequately cover the breadth of knowledge held by members of each community.

In total, 14 tacit knowledge questions were developed for the Hadza and 23 tacit knowledge questions were developed for the BaYaka (Table S2). If a participant stated that they knew how to complete a specific task, they were then asked (a) how they had learned the task (*did you learn by teaching, watching, or on your own?*) and (b) [*if teaching or watching*] from whom they had learned the task.

In order to measure explicit knowledge, SLL also developed a plant and animal questionnaire. The leaves, stems, fruit, or husk of edible plants and plants used for tool production were collected with the help of knowledgeable community members. The local names for animals, the ease of collecting these animals, and their multiple uses (e.g., for hide work, sinew for bow strings) were determined in focus groups with men. We then developed a list of 22 explicit knowledge questions for the Hadza and 26–28 explicit knowledge questions for the BaYaka (two plants were not found in 2017—Table S2). These questions involved plant press identification, identification of animals from drawings from the *Kingdon Field Guide to African Mammals* (Kingdon 2015)*,* and, in the case of the Hadza, a polaroid picture of a poison plant, for safety reasons. Based on our qualitative interviews with the BaYaka, we added two additional free-list questions on vines used for weaving carrying baskets, and for climbing. These activities occur daily among the BaYaka but are not performed by the Hadza.

A total of 88 Hadza between the ages of 6 and 65 (*M*_age_ = 31.7, 43% female) from three camps and 70 BaYaka between the ages of 8 and 65 (*M*_age_ = 31.5, 50% female) from four camps participated in the full questionnaire. All interviews were conducted with the help of interpreters. Among the Hadza, interviews were conducted in Swahili or Hadzane. Participants were encouraged to use Hadzane words for identifying plants and animals. Among the BaYaka, all interviews were conducted in Yaka (di.Aka).

#### Scoring

For a majority of the tacit knowledge portion of the questionnaire, participants scored 1 if they stated that they knew how to complete a given task, and 0 otherwise. The exception to this binary coding was in the domain of hunting and honey collecting, where participants were asked to free-list the species they had successfully collected. In such cases, free lists were preferred to yes/no questions because they adequately captured the high interpersonal variability expected in these domains, and because participant recall seemed highly accurate for these tasks. For the explicit knowledge portion of the questionnaire, participants scored 1 for every item named in the free-listing questions, and 1 for every correct answer in the plant and animal identification. Because many plants and animals have multiple names, a list of all names given for each of the plants and animals was generated and checked by interpreters for alternative names. Total scoring for each question can be found in Table S2.

#### Ranking Task Difficulty

In order to assess each community’s local perception of task difficulty, SLL took pictures of people participating in each task (Hadza: 14 tasks, BaYaka: 22 tasks—no ranking data available for Q23). Using these pictures, we led participants through a series of forced-pair comparisons. First, two pictures were pulled out of the deck and the participant was asked to select which task they considered the most difficult. The two pictures were then placed side by side in a row, from least to most difficult. A new picture was then taken from the deck and compared with each of the two activities in the row until its difficulty rank was established as either (a) intermediate in difficulty, and thus placed between the two pictures already laid out; (b) equivalent in difficulty, and thus placed vertically to one of the two pictures already laid out; (c) most difficult, and thus placed at the very right of the row; or (d) least difficult, and thus placed at the very left of the row. This process was repeated such that each new picture was compared with one roughly in the middle of the row until all pictures were ranked from least to most difficult. Participants were then asked to look over the row and make any changes they felt improved the ranking. The deck of pictures was shuffled before each participant began the forced-pair comparisons. A representative sample of 21 Hadza (*M*_age_ = 36.9, 52% female) and 23 BaYaka (*M*_age_ = 40.2, 57% female) adults participated in the ranking task. All participants ranked all activities. Activities that were ranked as tied in difficulty were assigned the median value of the rank placement they would have otherwise occupied (e.g., if three activities were tied for first place, all three would receive the median rank of 2). Intersubject rankings for tasks were reliable in both societies (Hadza: Cronbach’s α = 0.88, BaYaka: Cronbach’s α = 0.91, but see Figs. S4 and S5).

#### Age Estimation

Biological age was not known for participants. Thus, age was estimated following Crittenden et al. (2013) and others by ranking individuals from oldest to youngest—either within nuclear families or within sets of cousins. Age was estimated based on these ranks. For individuals under the age of 20, age was estimated at 1-year intervals. For individuals older than 20, age was estimated at 10-year intervals, from 25 onward (see Lew-Levy et al. 2020b for further details).

### Data Analysis

We took a Bayesian multi-response, multilevel modelling approach to data analysis. The model is multilevel because, across responses, our model estimates random effect parameters that represent, for instance, how highly each task was ranked, how likely each task was to be endorsed, how likely each task was to be learned via teaching as opposed to individual learning or observation, etc. These random effects are partially pooled across tasks to reduce overfitting. Similarly, we included random effect parameters for each participant to capture individual differences in subsistence knowledge and learning. Society and sex differences were also represented using random effects. This approach allows us to account for repeated observations and accommodate variation at different levels of the data (e.g., task, individual).

**Fig. 1:**
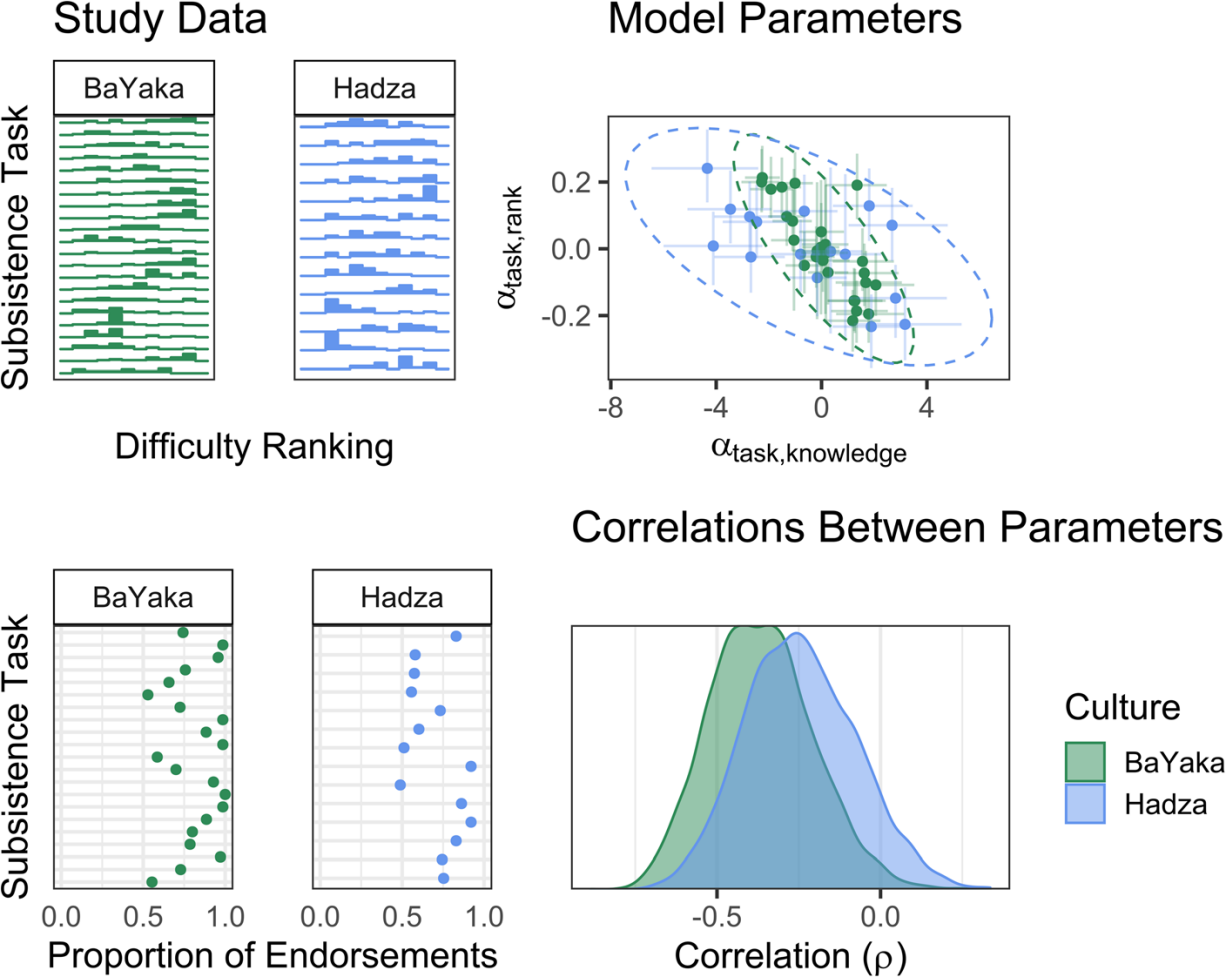
Visualization of how we move from raw data to assessing our hypotheses. Study data (left) feed into corresponding random effects for each task (top right), which are mapped onto a multivariate normal distribution. The correlation between random effects is estimated during model fitting (bottom right)

Our model is multi-response because five response variables are modelled simultaneously: (1) subjective ranking of task difficulty, (2) tacit/explicit subsistence knowledge, (3) learning method, (4) learning pathway, and (5) sex of transmitter. Each of these responses has its own submodel. By estimating correlations between sets of random effect parameters (i.e., the extent to which a task that is ranked highly is also likely to be endorsed; Fig. 1), a multi-response approach maintains the variance inherent to complex datasets. In doing so, our model avoids overconfidence in parameter estimates and false positives associated with including summary statistics (e.g., mean task rank difficulty) as model predictors in a conventional statistical approach (Loken and Gelman 2017).

We used a dynamical, life-history model of learning (Koster et al. 2020) to assess the effect of age on subsistence knowledge. For the learning/transmission responses, we included a second-order polynomial term for age to capture potential recall biases among older individuals (Fig. S6). Below we describe our model in greater detail, working through the submodels for each response.

- **H1:** Tasks ranked as more difficult are learned at a later age

Subsistence knowledge is a latent quantity elicited using questions about tacit and explicit knowledge. To test our hypothesis that more difficult tasks would be learned at later ages, we used a multi-response multilevel model that accounts for the diverse processes that lead to realized task performance.

**Fig. 2:**
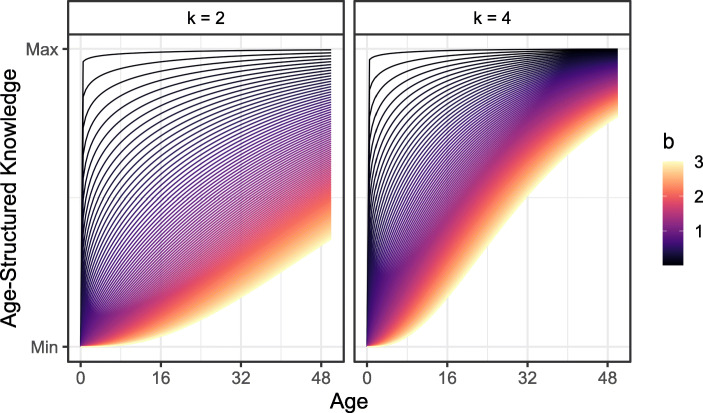
Hypothetical knowledge functions with constant = 2 (left) and = 4 (right) and *K* varying from 0.01 to 3. Depending on the combination of the two parameters, many different knowledge functions are possible, with the elasticity (*b*) determining the proportional increase in response to age

Following Koster et al. (2020), we use the von Bertalanffy (1934) growth model to represent knowledge accumulation across the lifespan. For a given age *x*, knowledge (*K*) increases at a learning rate *k*, proportional to how much age-structured knowledge there is left to learn (Fig. 2). Knowledge is modified by an elasticity parameter *b*, which determines the magnitude of knowledge gain in response to learning:

$$ \frac{dK}{dx}=k\left(1-K(x)\right) $$

$$ K(x)={\left(1-{\mathrm{e}}^{- kx}\right)}^b $$

We partially pooled information across different tasks using random effects, so that each task has a unique *k* and *b* parameter. In principle, the rate of learning and knowledge elasticity could also vary between individuals. Because the data in this study are not longitudinal, it is not possible to estimate individual differences in learning rates. We also did not know the precise age at which proficiency in subsistence tasks were first acquired. Instead, we had an estimated age at the time of interview. If a participant endorsed a task, this means that they learned it sometime between their birth and their current age. In the nomenclature of survival analysis, these data are interval-censored. Age of skill acquisition was thus probabilistically inferred by its presence or absence among participants of varying ages.

The learning parameters were defined as:

$$ k={\mathrm{e}}^{\left({\upalpha}_{\left[k\right]}+{\upalpha}_{\left[\mathrm{task},k\right]}\right)} $$

$$ b={\mathrm{e}}^{\left({\upalpha}_{\left[b\right]}+{\upalpha}_{\left[\mathrm{task},b\right]}\right)} $$

The next component of this function is the elasticity of knowledge, denoted η, which determines how much age-structured learning affects task performance. We allowed η to vary for each task, with a different average elasticity for tacit and explicit knowledge tasks. As η approaches 0, accumulated knowledge has no effect on task performance. We assumed that η is strictly positive:

$$ \upeta ={\mathrm{e}}^{\left({\upalpha}_{\left[\upeta \right]}+{\upalpha}_{\left[\upeta\ \mathrm{explicit}\right]} explicit+{\upalpha}_{\left[\mathrm{task},\upeta \right]}\right)} $$

In addition to variation due to age and task difficulty, we expected variation due to sex, society, and individual differences in task performance. We accounted for this variation by estimating baseline differences in task performance independent of age for each task, denoted α. The inclusion of these random effects allowed us to account for multiple data-generating processes in our study. For example, it is not the case that females have a different learning function (*K*) than males for bow hunting—instead, females never learn this task because they specialize in other domains. Rather than try to estimate sex differences in the learning process of subsistence tasks, we instead modelled sex differences in baseline task performance, independent of age. This means that the model effectively averages over the effect of sex on age of acquisition while accounting for the fact that many tasks are sex-specific regardless of age.

For the identification tasks, we included random effects for each species. The individual difference parameters capture overall performance and are estimated separately for tacit and explicit knowledge tasks so we can assess the correlation between the two (see model details for H5). To account for covariance between subsistence domains (e.g., hunting and identification of animals), we also modelled residual correlations between each task with observation-level random effects, denoted by α_[obs]_.

$$ {\displaystyle \begin{array}{c}\ \\ {}\ \begin{array}{c}\upalpha ={\upalpha}_0+{\upalpha}_{\left[\mathrm{explicit}\right]}\mathrm{explicit}+{\upalpha}_{\left[\mathrm{freelist}\right]}\mathrm{freelist}+{\upalpha}_{\left[\mathrm{sex}\ast \mathrm{society},\mathrm{knowledge}\right]}\\ {}+{\upalpha}_{\left[\mathrm{id},\mathrm{knowledge}\right]}\mathrm{tacit}+{\upalpha}_{\left[\mathrm{id},\mathrm{knowledge}\left(\mathrm{explicit}\right)\right]}\mathrm{explicit}+{\upalpha}_{\left[\mathrm{task},\mathrm{knowledge}\right]}\\ {}+{\upalpha}_{\left[\mathrm{task},\mathrm{knowledge}\left(\mathrm{sex}\right)\right]}\mathrm{sex}+{\upalpha}_{\left[\mathrm{plant}\right]}+{\alpha}_{\left[\mathrm{animal}\right]}+{\upalpha}_{\left[\mathrm{obs},\mathrm{task}\right]}\end{array}\end{array}} $$

$$ {\upalpha}_{\left[\mathrm{obs}\right]}\sim \mathrm{MVNormal}\left(\left[\begin{array}{r}0\\ {}\vdots \\ {}{N}_{\mathrm{obs}}\end{array}\right],{\boldsymbol{\Sigma}}_{\mathrm{obs}}\right) $$

$$ {\boldsymbol{\Sigma}}_{\mathrm{obs}}=\left[\begin{array}{rrrr}{\upsigma}_{\left[\mathrm{task}=1\right]}& 0& 0& 0\\ {}0& {\upsigma}_{\left[\mathrm{task}=2\right]}& 0& 0\\ {}0& 0& \ddots & 0\\ {}0& 0& 0& {\upsigma}_{\left[{N}_{\mathrm{task}}\right]}\end{array}\right]{\boldsymbol{\Omega}}_{\mathrm{obs}}\left[\begin{array}{rrrr}{\upsigma}_{\left[\mathrm{task}=1\right]}& 0& 0& 0\\ {}0& {\upsigma}_{\left[\mathrm{task}=2\right]}& 0& 0\\ {}0& 0& \ddots & 0\\ {}0& 0& 0& {\upsigma}_{\left[{N}_{\mathrm{task}}\right]}\end{array}\right] $$

Where **Ω**_obs_ is a *N*_task_ × *N*_task_ correlation matrix.

The overall expected value μ is:

$$ \upmu ={K}^{\upeta}\ast {\mathrm{e}}^{\upalpha} $$

For the tacit knowledge and identification tasks, responses are modelled using the Bernoulli distribution and the link function from Koster et al. (2020). The probability of an endorsement/correct identification is thus:

$$ \Pr \left(y=1\right)=2\left(\frac{{\mathrm{e}}^{\upmu}}{1+{\mathrm{e}}^{\upmu}}-\frac{1}{2}\right) $$

For the free-list tasks, we modelled the responses using the Negative Binomial Distribution because the data are counts with potential overdispersion (ϕ):

$$ {y}_{\left[\mathrm{freelist}\right]}\sim \mathrm{NegativeBinomial}\ \left(\frac{\upmu^2}{\upphi -\upmu},\frac{\upphi -\upmu}{\upphi}\right) $$

Thus, task performance was modelled as the product of a latent age-structured learning function (*K*) and a linear model (α). By partitioning these components, we can compare across tasks that have different response types (binary, free list) and disentangle the life history of learning from the linear distribution of a given response. In other words, we separate the pace of learning from the absolute values of each response, allowing for comparison across heterogenous data structures. Throughout, we use the term “age-structured learning” as a shorthand for the way that response performance increases as a function of age.

- **H2:** Tasks ranked as more difficult are more likely to be learned via teaching

To test the hypothesis that more difficult tasks are more likely to be learned via teaching, we employed a multilevel categorical model of *K* = 3 categories (and thus *K* − 1 equations) representing the probability of observational learning, teaching, and individual learning (see Koster and McElreath 2017 for more details on multilevel categorical models).

We model variation between tasks and between individuals using random effects. These are correlated with the corresponding parameters in our other outcomes (knowledge, transmission pathway, etc.). We also included sex and societal differences in transmission, and a second-order polynomial term on age-at-interview to account for potential recall bias among older individuals. The multi-logit probability *p*_[method]_ of a given transmission method *k ϵ* 1:*K* − 1 is:

$$ {p}_k={\upalpha}_{\left[{\mathrm{method}}_k\right]}+{\upalpha}_{\left[\mathrm{task},{\mathrm{method}}_k\right]}+{\upalpha}_{\left[\mathrm{sex}\ast \mathrm{society},{\mathrm{method}}_k\right]}+{\upalpha}_{\left[\mathrm{id},\mathrm{male}\right]}+{\upbeta}_{\left[\mathrm{age},{\mathrm{method}}_k\right]}\mathrm{age}+{\upbeta}_{\left[{\mathrm{age}}^2,{\mathrm{method}}_k\right]}{\mathrm{age}}^2 $$

The softmax function converts the vector *p*_[method]_ from the multi-logit scale to a simplex of probabilities:

$$ {y}_{\left[\mathrm{method}\right]}\sim \mathrm{Categorical}\ \left(\frac{{\mathrm{e}}^p}{\sum \limits_{k=1}^K{\mathrm{e}}^p}\right) $$

- **H3:** Tasks ranked as more difficult are more likely to be learned obliquely

To test the hypothesis that more-difficult tasks are more likely to be learned obliquely, we organized responses into three categories: horizontal, oblique, and vertical (Table 2). We define both parent- and grandparent-to-offspring transmission as vertical because it follows the same path of inheritance as do genes. Transmission from aunts, uncles, and other kin who are not direct ancestors is defined as oblique because, although they share genes, the focal children are not genetically descended from those kin. The structure of this multilevel categorical submodel is identical to that of the transmission method described previously.

- **H4:** Same-sex transmission is more likely than opposite-sex transmission

**Table 2:** Responses to the question “Whom did you learn from?” categorized by learning pathway

Pathway	Response
Vertical	Father, mother, both parents, grandfather, grandmother, both grandparents
Horizontal	Sibling(s), cousin(s), friend(s), spouse, youth, brother-in-law, older child/children, older young man/men, adolescent(s)
Oblique	Aunt(s), uncle(s), older man/men, older relative(s), elder(s), best hunter(s), man/men, woman/women, person/people

Notes: We excluded a single nomination of “farmer” because it was not known if this individual was in the same age cohort as the participant. If participants named more than one category of people from whom they learned, we categorized the pathway as it related to the closest kinship category (e.g., father and uncle → vertical)

To test the hypothesis that same-sex transmission is more likely than opposite-sex transmission, we organized responses into two categories: male or female. If participants named teachers of both sexes (e.g., parents, grandparents), the model marginalized over “Male” and “Female” with equal probability. The log-odds of male transmission *p*_[male transmission]_ were defined as:

$$ {\displaystyle \begin{array}{c}{p}_{\left[\mathrm{male}\ \mathrm{transmission}\right]}={\upalpha}_{\mathrm{male}}+{\upalpha}_{\left[\mathrm{sex}\ast \mathrm{society},\mathrm{male}\right]}+{\upalpha}_{\left[\mathrm{id},\mathrm{male}\right]}+{\upalpha}_{\left[\mathrm{task},\mathrm{male}\right]}\\ {}+{\upbeta}_{\left[\mathrm{age},\mathrm{male}\right]}\mathrm{age}+{\upbeta}_{\left[{\mathrm{age}}^2,\mathrm{male}\right]}{\mathrm{age}}^2\end{array}} $$

$$ \Pr \left({y}_{\left[\mathrm{male}\ \mathrm{transmission}\right]}=1\right)=\frac{1}{\left(1+{\mathrm{e}}^{-{p}_{\left[\mathrm{male}\ \mathrm{transmission}\right]}}\right)} $$

- **H5:** Tacit and explicit knowledge are positively correlated

To assess whether individuals performed similarly on both tacit and explicit tasks, we looked at the correlation between individual-level random effects (*α*_[id, knowledge]_, *α*_[id, knowledge(explicit)]_) for performance on the two types of task. We first fit a model without any sociodemographic information (age, sex), which we expected to account for some individual variation in the tasks. Specifically, we modelled individual-level random effects on task performance as a bivariate normal distribution:

$$ {\upalpha}_{\left[\mathrm{id},\mathrm{knowledge}\right],}{\upalpha}_{\left[\mathrm{id},\mathrm{knowledge}\left(\mathrm{explicit}\right)\right],}\sim \mathrm{MVNormal}\left(\left[\begin{array}{r}0,\\ {}\vdots, \\ {}{N}_{\mathrm{id},}\end{array}\begin{array}{r}0\\ {}\vdots \\ {}{N}_{\mathrm{id}}\end{array}\right],{\boldsymbol{\Sigma}}_{\mathrm{id}}\right) $$

$$ {\boldsymbol{\Sigma}}_{\mathrm{id}}=\left[\begin{array}{c}{\upsigma}_{\left[\mathrm{id},\mathrm{knowledge}\right]},\kern3.25em 0\\ {}\ 0,{\upsigma}_{\left[\mathrm{id},\mathrm{knowledge}\left(\mathrm{explicit}\right)\right]}\ \end{array}\right]{\boldsymbol{\Omega}}_{\mathrm{id}}\left[\begin{array}{c}{\upsigma}_{\left[\mathrm{id},\mathrm{knowledge}\right]},\kern3em 0\\ {}\ 0,{\upsigma}_{\left[\mathrm{id},\mathrm{knowledge}\left(\mathrm{explicit}\right)\right]}\ \end{array}\right] $$

where **Ω**_id_ is a 2 × 2 correlation matrix.

We also included these parameters in our full multi-response model to examine whether there was residual correlation between individual tacit and explicit performance after accounting for age and sex.

#### Rank Data

We opted for a simple Gaussian approximation of participants’ task difficulty ranking. Although there are more-sophisticated ways to model subjective ranking data (e.g., Thurstonian item-response models; Giles et al. 2018; Maydeu-Olivares 1999), our hypotheses about ranks concerns only the average for each task. Our submodel was thus:

$$ {y}_{\left[\operatorname{rank}\right]}\sim \mathrm{Normal}\left({\upalpha}_{\left[\mathit{\operatorname{rank}}\right]}+{\upalpha}_{\left[\mathrm{task},\operatorname{rank}\right]},{\upsigma}_{\left[\operatorname{rank}\right]}\right) $$

#### Measurement Error for Participant Age

Given the uncertainty in age estimation, we replaced the raw point estimates (age_obs_) with a Gaussian measurement error model (age_est_) with a standard deviation equal to half of the interval range (0.5 years for those under 20, 5 years for those above 20).

$$ {\mathrm{age}}_{\mathrm{obs}}\sim \mathrm{Normal}\left({\mathrm{age}}_{\mathrm{est}},{\upsigma}_{\mathrm{obs}}\right) $$

#### Model Fitting

All analyses were run in R 3.6.1 (R Core Team 2013) and all models were fit using the RStan package (Stan Development Team 2020), which fits Bayesian models using Hamiltonian Markov Chain Monte Carlo. We employed regularizing priors for all parameters to reduce overfitting and facilitate model convergence. Markov chain convergence was assessed using standard diagnostics (number of effective samples, the Gelman-Rubin diagnostic, and visual inspection of trace plots).

## Results

- **H1:** Tasks ranked as more difficult are learned at a later age

**Fig. 3:**
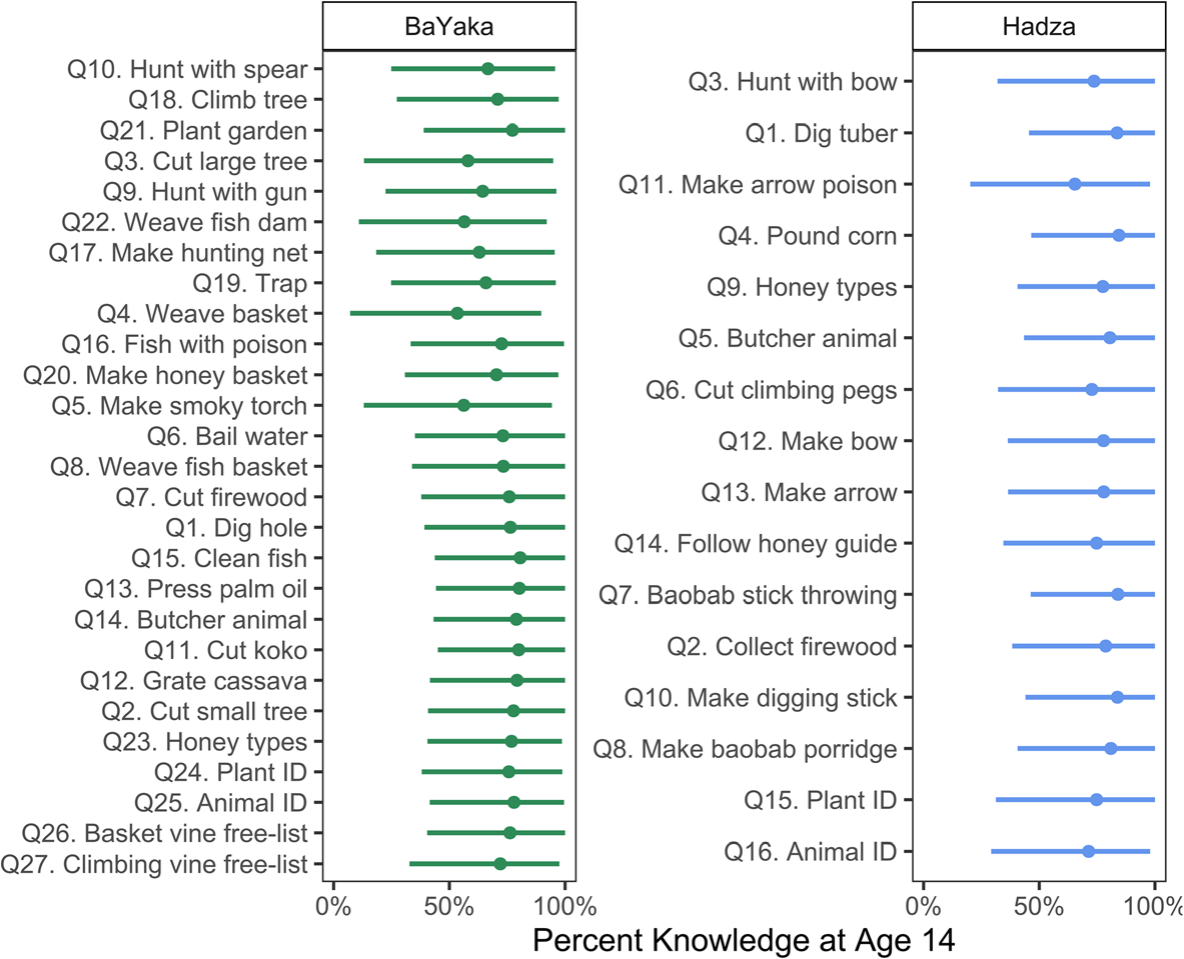
Cumulative percentage of age-structured learning by age 14 for each task. Points represent median posterior *K* estimates and bars represent 90% HPDI (right). Tasks are sorted by median difficulty ranking

**Fig. 4:**
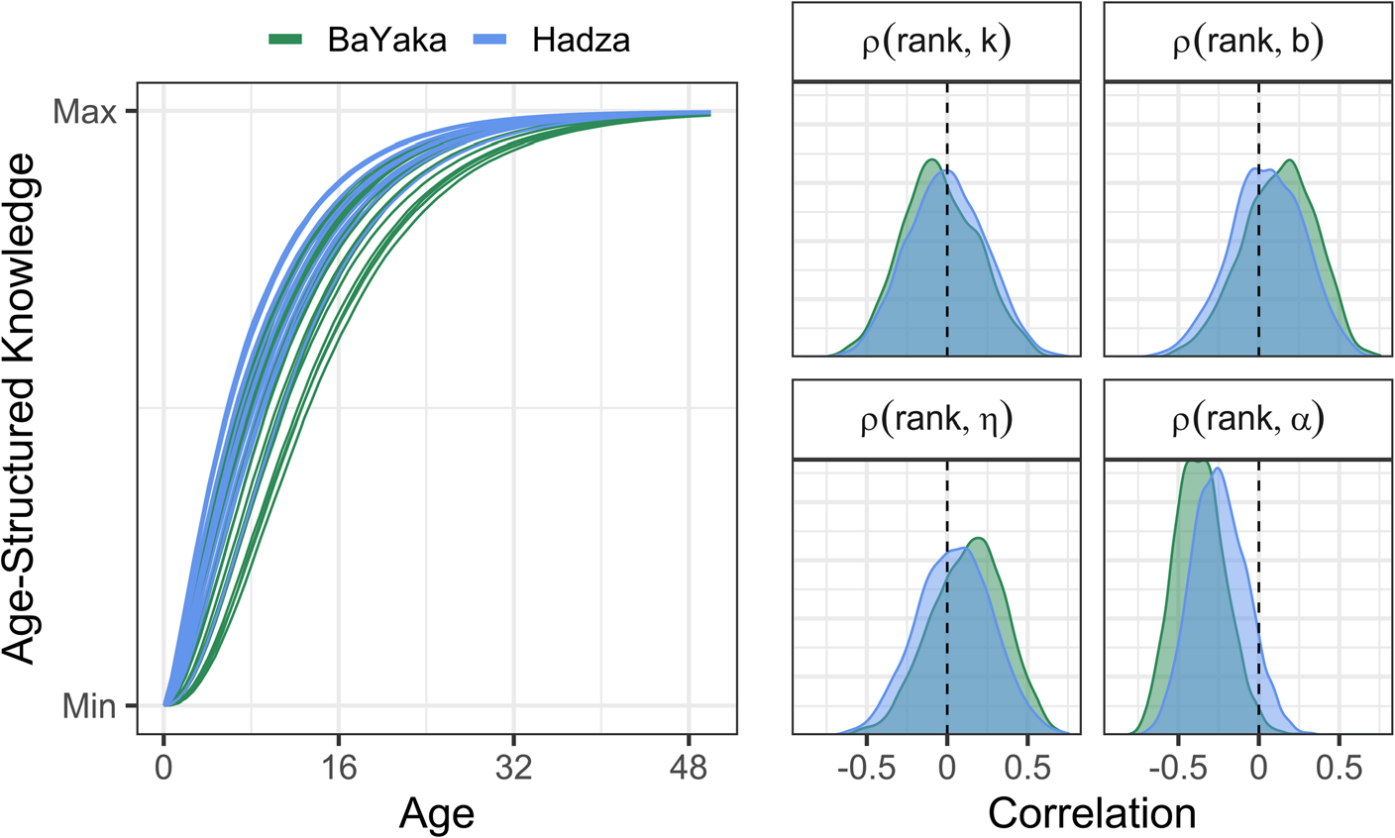
(Left) Age-structured knowledge across the lifespan. Each line represents the posterior median *K* function for a single subsistence task. The shape of learning is similar across tasks and between societies, with most age-structured learning occurring by the age of 14. (Right) Posterior correlations between task difficulty ranking and the rate of learning (*k*), the elasticity of learning on knowledge (*b*), the elasticity of knowledge on task performance (η), and age-independent task performance (α). Although difficulty rankings were not correlated with any of the age-structured learning parameters, tasks ranked as more difficult generally had lower performance (i.e., fewer individuals knew how to do the task), independent of age

By age 14, participants reported having achieved 74% (90% HPDI = [39%, 96%]) of their age-structured learning for a typical task (Figs. 3 and 4, S1 and S2). Although some types of subsistence knowledge are estimated to accumulate earlier or later, prediction intervals for all tasks overlap. This lack of variation limits our ability to assess H1.

The relevant parameters to assess H1 are the correlations (ρ) between the *k* (rate of learning), *b* (elasticity of learning on knowledge), and *η* (elasticity of knowledge on performance) random effects across different tasks and the random effects representing the mean subjective difficulty rank of each task. But virtually all these correlations were small (the median ρ ranged from −0.06 to 0.14) and with high uncertainty (Figs. 4 and S7). Thus, H1 was not supported. However, tasks ranked as more difficult exhibited lower average performance (i.e., fewer individuals knew how to do the task) independent of age (median ρ_[rank, task(α)]_ = −0.37, 90% HPDI = [−0.62, −0.11] for BaYaka; ρ_[rank, task(α)]_ = −0.25 [−0.53, 0.02] for Hadza). Median posterior correlations between task-specific random effects for all five submodels can be found in Tables S3 and S4.

- **H2:** Tasks ranked as more difficult are more likely to be learned via teaching

Teaching was the most common method of subsistence learning for the BaYaka, whereas observation was the most common method for the Hadza (Table 3; Fig. S8). Individual learning was the least common method in both societies, and the sexes had similar learning profiles within societies. The interindividual variance for learning method was high in both societies, particularly among Hadza (Fig. S3). There was a small positive correlation between task difficult ranking and the probability of transmission via teaching for both BaYaka (median ρ_[rank,teach]_ = 0.16, 90% HPDI = [−0.19,0.51]) and Hadza (median ρ_[rank,teach]_ = 0.25 [−0.08,0.61]). Thus, H2 was weakly supported.

- **H3**: Tasks ranked as more difficult are more likely to be learned obliquely

**Table 3:** Probability of each learning method for BaYaka females and males, and Hadza females and males

	Observation	Teaching	Individual
BaYaka Females	19.50% [5.89,36.73]	78.64% [60.62,93.77]	1.69% [0.29,3.69]
BaYaka Males	21.99% [7.25,38.76]	76.42% [57.99,91.21]	1.45% [0.31, 3.08]
Hadza Females	64.76% [47.07,82.40]	30.18% [12.81,48.35]	4.67% [1.86,8.12]
Hadza Males	69.25% [53.53,82.66]	25.02% [11.72,40.07]	5.15% [2.45,8.51]

Oblique learning was the least common transmission pathway in both societies (Table 4; Fig. S9), although it was slightly higher among the Hadza than the BaYaka, particularly for Hadza males. We did not find a consistent correlation between task difficulty ranking and the probability of oblique transmission (BaYaka: ρ_[rank,oblique]_ = 0.1, 90% HPDI = [−0.27,0.49]; Hadza: ρ_[rank,oblique]_ = −0.06 [−0.40,0.32]). Thus, H3 was not supported.

- **H4:** Same-sex transmission is more likely than opposite-sex transmission

**Table 4:** Probability of each learning pathway for BaYaka females and males, and Hadza females and males

	Horizontal	Oblique	Vertical
BaYaka Females	1.03% [0.07,2.77]	0.08% [0,0.41]	98.78% [97.09,99.91]
BaYaka Males	0.11% [0,0.41]	0.23% [0,0.99]	99.58% [98.74,99.98]
Hadza Females	0.61% [0.01,2.04]	1.90% [0.24,4.26]	97.21% [94.36,99.50]
Hadza Males	1.04% [0.04,2.89]	4.18% [1.14,7.74]	94.47% [90.27,98.10]

**Fig. 5:**
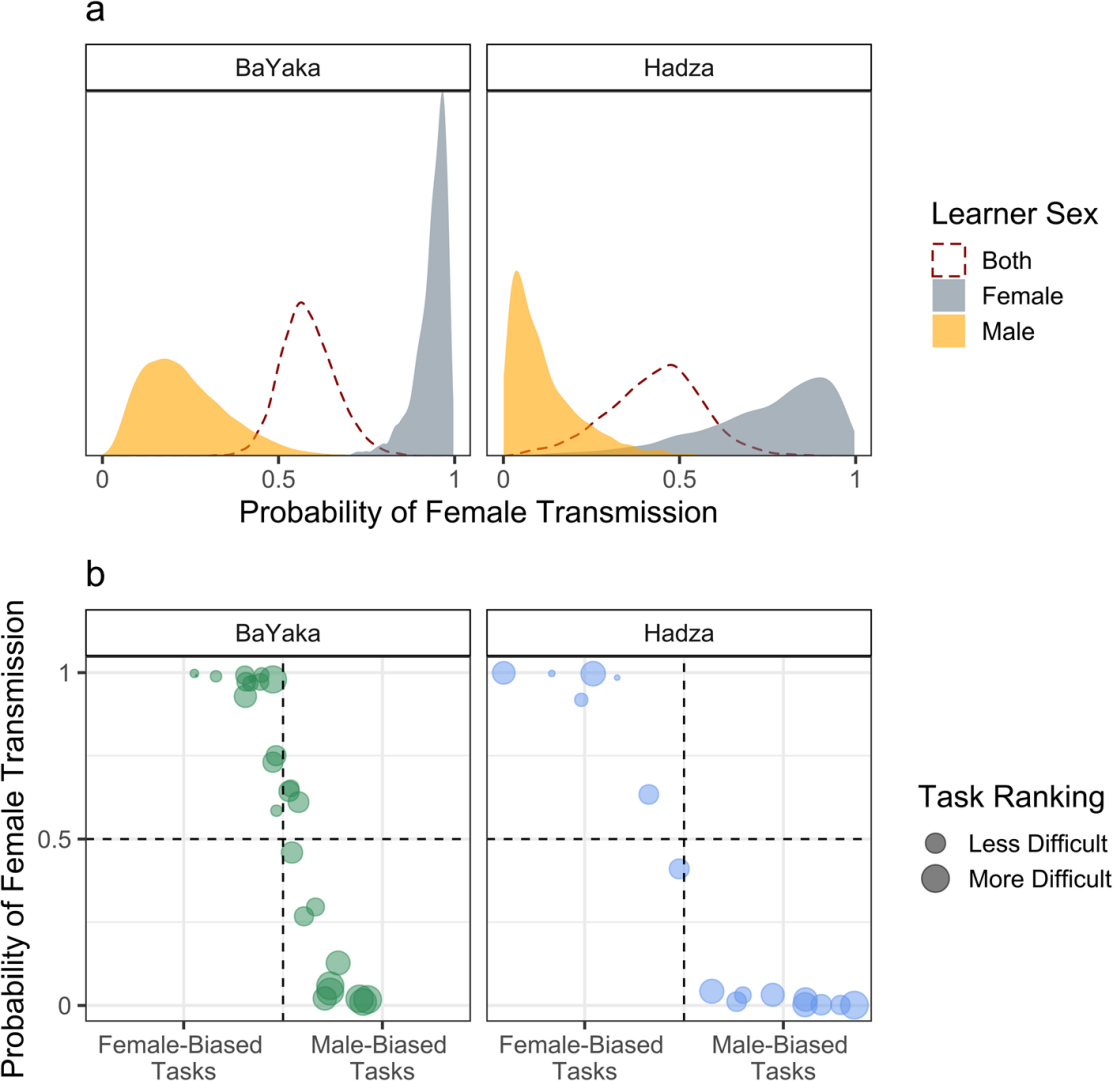
**a** The posterior probabilities of female transmission via observation or teaching to male and female learners in each society. **b** Median posterior probabilities of female transmission for each subsistence task, plotted against the degree of differentiation in task performance by sex (α_[task,male]_). Tasks with the greatest and least amount of differentiation by sex are annotated for each society. Point size is proportional to the median task difficulty rankings

Consistent with expectations, same-sex transmission was more likely than opposite-sex transmission among BaYaka (median Pr[Same Sex Transmission] = 85.99%, 90% HPDI = [78.05%, 93.38%]) and Hadza (Pr[Same Sex Transmission] = 82.39%, [70.35%,93.62%]) (Fig. 5a). Thus, H4 was supported. Additionally, BaYaka women appeared to be responsible for more knowledge transmission than men overall (Pr[Female Transmission] = 57.73% [45.71,70.68]), whereas the opposite was true for Hadza (Pr[Female Transmission] = 43.62% [19.03,62.85]). However, these estimates average over the variation across tasks. Figure 5b shows that the sexual division of transmission corresponds closely to the sexual division of subsistence labor (i.e., tasks where there was differentiation by sex in performance/knowledge). In both societies, males tended to transmit male-specific tasks, and vice versa (median ρ_[task(male),task(female transmission)]_ = −0.60, 90% HPDI = [−0.81,−0.39] for BaYaka; ρ_[task(male),task(female transmission)]_ = −0.54 [−0.77,−0.27] for Hadza). Moreover, tasks ranked as more difficult were more likely to be transmitted by men among the BaYaka (ρ_[rank,task(female transmission)]_ = −0.33, 90% HPDI = [−0.58, −0.09]) but not the Hadza (ρ_[rank,task(female transmission)]_ = −0.11, 90% HPDI = [−0.40, 0.17]).

- **H5:** Tacit and explicit knowledge are positively correlated

**Fig. 6:**
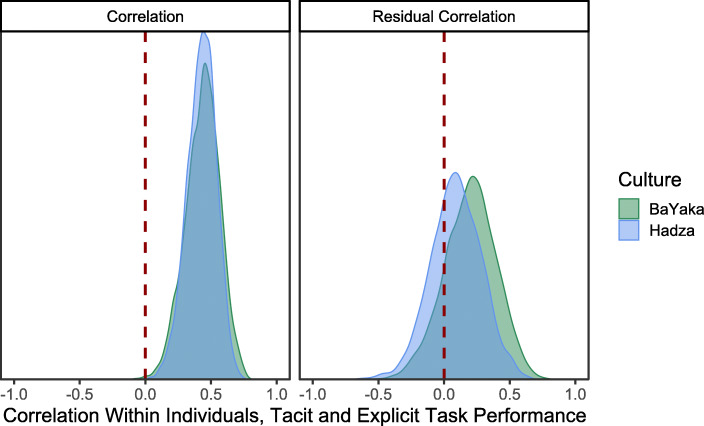
The posterior correlations between individual performance on tacit and explicit knowledge tasks, without controlling for age and sex (**left**) and controlling for age and sex (**right**). Individuals who perform higher on tacit knowledge tasks also tend to perform higher on explicit knowledge tasks in both societies. This correlation is mediated by age and sex differences between individuals (residual correlation)

To assess the consistency of tacit and explicit knowledge, we looked at the residual correlation between the individual random effects conditional on age and sex. We found a moderate correlation between individual’s performance on the tacit and explicit knowledge tasks among BaYaka (ρ_[id tacit, id explicit]_ = 0.44 [0.22, 0.65]) and Hadza (ρ_[id tacit, id explicit]_ = 0.43 [0.26, 0.62]). The residual correlations between these tasks were smaller (BaYaka = 0.19 [−0.13, 0.52], Hadza = 0.09 [−0.24,0.37]), as anticipated given the mediating role of age and sex on individual differences (Fig. 6). H5 was supported overall. Additionally, the individual variation in tacit knowledge tasks (median σ_[id,tacit]_ = 0.67, 90% HPDI = [0.49, 0.83] for BaYaka; σ_[id,tacit]_ = 0.59 [0.46, 0.75] for Hadza) was greater than explicit knowledge tasks (σ_[id,explicit]_ = 0.2 [0.07, 0.32] for BaYaka; σ_[id,explicit]_ = 0.38 [0.12, 0.53] for Hadza).

## Discussion

This study used interview data to examine the relationship between task difficulty and knowledge acquisition among Hadza and BaYaka foragers. Contrary to our hypothesis, we found that task difficulty was not strongly correlated with age of acquisition, and that participants reported knowing how to perform most tasks by age 14. This finding runs contrary to those from rural Fijians and Tsimane forager-horticulturalists, where more-difficult tasks were acquired at later ages (Kline et al. 2013; Schniter et al. 2015). There are several potential explanations for these differing results. First, our findings are consistent with previous research on Hadza and BaYaka children’s foraging knowledge, which shows that most aspects of subsistence are acquired by early adolescence (Crittenden 2016b; Gallois et al. 2015; Hewlett and Cavalli-Sforza 1986). Forager children may acquire subsistence knowledge in early life because they can readily observe, and are encouraged to participate in, most food production activities (see Lew-Levy et al. 2017 for review). Indeed, both Hadza and BaYaka children are active foragers from an early age, even as their foraging returns vary (Crittenden et al. 2013; Hagino and Yamauchi 2016). Thus, more data from younger children may be needed to properly evaluate our hypothesis. Further, unlike subsistence skills, conventional skills, such as cultural norms and ceremonial knowledge, are less readily observable (e.g., Lewis 2015) and thus may take longer to master. Among the Tsimane, for example, subsistence skills were acquired early, between the ages of 13 and 16, whereas storytelling skills were acquired later, between ages 16 and 23 (Schniter et al. 2015). Future studies will investigate how the acquisition of subsistence knowledge compares with that of conventional skills.

Methodological differences between our study design and those by Kline et al. (2013) and Schniter et al. (2015) may also explain our differing findings with regard to age of knowledge acquisition. In both of those studies, participants reported the age at which they gained basic competency at a given task (Tsimane) or the age at which children ought to start learning a given task (Fijian). However, since the BaYaka and Hadza rarely know their ages in years, recalling age of acquisition is difficult in these populations. Furthermore, since we did not collect objective measures of skill (e.g., daily food returns), it is possible that younger children overestimate their skill when answering some questions (Bjorklund and Green 1992). However, considering our finding that tacit and explicit knowledge were strongly correlated, even when adjusting for age, this overestimation is likely minimal. Despite these limitations, and consistent with previous findings from forager societies, our results suggest that, for most subsistence tasks, knowledge acquisition occurs early on, even though children refine their knowledge and skill with age.

Teaching was an important mechanism for cultural transmission among both the Hadza and BaYaka. Teaching is a costly activity, and may have evolved to facilitate the transmission of opaque and difficult-to-acquire knowledge (Csibra and Gergely 2006, 2011; Kline 2015). Although weak, our results suggest that tasks ranked as more difficult were more likely to be taught in both populations. The weakness of our results may be due to the readily observable nature of subsistence tasks. Conventional skills are less readily observable to children, and thus, teaching may be more common in these domains (Kline 2015; Lew-Levy et al. 2018). For example, among the Inuit, adults engage children in games to teach them kinship terms (Guemple 1988). Among the San, parents encourage infants as young as 8 months old when they hand objects to others (Bakeman et al. 1990). In doing so, adults promote sharing, a foundational schema in this population. Thus, although we found teaching to be marginally important for the acquisition of difficult subsistence tasks, it may be central to the acquisition of opaque cultural norms and skills.

Our results did not support the hypothesis that difficult tasks would be acquired via oblique transmission. We found that most subsistence tasks were learned in early life. Previous research in diverse populations has shown that vertical transmission is common in early life (e.g., Eyssartier et al. 2008; Kline 2015; Reyes-García et al. 2016). Further, in a cultural knowledge system at or near equilibrium, nearly all individuals in a community will share the same knowledge, resulting in little oblique transmission following vertical transmission (Henrich and Broesch 2011). Thus, the oblique transmission of subsistence tasks may be rare among the Hadza and BaYaka because these tasks are performed by most of the population. Several aspects of our study design may have also limited our ability to detect the importance of oblique transmission in our analysis. First, oblique transmission may be primarily used to refine previously acquired knowledge rather than acquire new knowledge forms (Reyes-García et al. 2016). For example, although an individual may learn to hunt from their father or weave baskets from their mother in early life, they may also seek out expert adults to learn specialized aspects of these tasks, such as innovative basketry design (Dira and Hewlett 2016; Hewlett 2013, 2016, 2021; Hewlett and Hewlett 2012). Several studies also suggest that recall data biases respondents toward nominating categories of individuals from whom knowledge transmission is expected instead of those from whom it actually occurs (e.g., Aunger 2000; Dira and Hewlett 2016). Since participants were asked to nominate only one individual from whom they had acquired a skill, vertical transmission was likely overemphasized, and oblique transmission underemphasized, in our sample.

As in other studies (e.g., Boyette and Hewlett 2017; Demps et al. 2012; Flannery 1953; Hagen et al. 2016; Hewlett 2012; Hewlett and Cavalli-Sforza 1986; Lew-Levy et al. 2017), we found strong evidence for same-sex transmission. Since all human populations maintain a sexual division of labor, learning from same-sex individuals likely facilitates the acquisition of sex-typical tasks. Interestingly, we found that tasks ranked as more difficult were more likely to be transmitted by men among the BaYaka, but not among the Hadza. As noted earlier, the Hadza maintain a stronger sexual division of labor than the BaYaka, with Hadza men exclusively hunting and Hadza women primarily plant collecting, whereas BaYaka men and women participate in aspects of both hunting and collecting. Thus, our findings may reflect a tension between the sexual division of labor and the sexual division of teaching labor. When knowledge is shared between the sexes, such as among the BaYaka, women are primarily responsible for transmitting easier tasks, likely because such knowledge is transmitted earlier in life, when women and children occupy the same social spaces, and when women are the main beneficiaries of children’s work (Bradley 1993). When knowledge is sex-specific, such as among the Hadza, transmission can only occur within same-sex dyads, and thus men and women may take on a more equal share of the teaching labor for both easier and more-difficult tasks.

Finally, this paper also examined cross-cultural differences in social learning. First, both the Hadza and BaYaka reported low rates of horizontal transmission. This finding counters our observational research, which showed high rates of child-to-child transmission in both societies (Lew-Levy et al. 2020b). Since much horizontal transmission occurs within the multi-age mixed-sex playgroup, where children seamlessly transition from play to work activities (Crittenden 2016b; Lew-Levy et al. 2019, 2020a), it is likely that children do not consider experiences in the playgroup as salient moments of knowledge transmission even though they make up a large proportion of children’s time allocation in both societies. As well, our interviews focused on adult domains of knowledge; children are more likely, however, to report learning horizontally when asked about subsistence tasks conducted by children only, such as rat hunting among the Baka (Gallois et al. 2017). Further, we found that social learning primarily occurred via teaching among the BaYaka, whereas Hadza participants primarily reported learning via observation. As noted earlier, Hadza adults do not view themselves as the primary teachers of children (Crittenden 2016b; Lew-Levy et al. 2019). Instead, they facilitate autonomous participation in subsistence tasks by making children functional tools. Among the BaYaka, adults report teaching as an important parental role (Boyette et al. 2019), and families often forage together (Lew-Levy et al. 2019). In our observational research (Lew-Levy et al. 2020a, b), we suggest that these socialization differences may result in lower rates of adult teaching among Hadza children and adolescents than among the BaYaka. The interview data from the present paper further support this claim.

## Limitations

This paper has several limitations. First, the rankings included in our models did not differentially account for strength-based vs. skill-based difficulty (Kramer 2011). Theoretically, participation in strength-based activities, such as collecting firewood, may be delayed until an individual is strong enough to perform the task successfully, after which knowledge acquisition is relatively rapid (Bock 2002). Skill-based tasks, such as hunting, may be more contingent on previous learning experience and thus may take longer to learn (Walker et al. 2002). Although we originally planned to collect ranked data on difficulty of both strength-based and skill-based tasks, this process was too time consuming and tedious for participants. Further, participants themselves did not distinguish between strength- and skill-based task difficulty, making the rankings redundant. Thus, a simplified overall difficulty measure was collected instead.

Second, although the tacit knowledge task aimed at assessing participants’ previous experience, some of the participants may have reported knowledge of performing a task without ever having actually completed the task themselves. However, several lines of evidence suggest that this was not the case. Participant responses reflected the sexual division of labor in each society, despite the fact that most participants have likely observed, and thus know the steps involved in, nearly all subsistence tasks. Similarly, children sometimes reported not knowing how to perform a task but stated that they were in the process of learning. Finally, we sometimes cross-checked children’s tacit knowledge responses with their parents. Parents overwhelmingly corroborated children’s accounts by recounting the specific times that children had participated in the task in question. For these reasons, we take our knowledge questionnaire as adequately measuring participants’ tacit knowledge.

Third, whereas previous studies suggest that teaching is more likely to occur between closely related kin (Boyette and Hewlett 2017; Kline et al. 2013), including in the populations surveyed here (Lew-Levy et al. 2020a, b), we were not able to investigate these trends because of the low frequency of horizontal and oblique transmission reported. Nonetheless, the dual importance of teaching and of vertical transmission supports the view that teaching is more likely to come from kin than non-kin.

Finally, several studies conducted among Aka and Chabu foragers demonstrate that adolescents preferentially learn complex skills from individuals they consider to be especially competent teachers (Dira and Hewlett 2016; Hewlett 2013, 2016, 2021). Although we found only a weak relationship between teaching and skill complexity, teacher quality may be an important, and unaccounted for, factor in the transmission of complex knowledge via teaching.

## Conclusion

Using interview data from two forager societies—the Hadza of Tanzania and the BaYaka of the Republic of Congo—this paper examined the effect of task difficulty on age of knowledge acquisition, rates of teaching, and rates of oblique transmission in the domain of subsistence. Our findings suggest that task difficulty is not strongly related to age of acquisition or oblique transmission. Children knew how to perform most explicit and tacit knowledge tasks by age 14, though task-specific skill likely continues to increase with age. Further, teaching was marginally more likely to occur for more difficult tasks. We also found cross-cultural variation in how and from whom learning occurred. Although same-sex transmission was normative in both societies, BaYaka men were more likely to teach difficult tasks than BaYaka women. No strong sex differences in the transmission of easy vs. difficult tasks were found among the Hadza. This finding potentially reflects cross-cultural variation in the sexual division of subsistence and teaching labor. In addition, we found that the Hadza reported learning primarily via observation, whereas BaYaka participants reported learning primarily via teaching. We argue that these differences can be explained by examining the socialization practices of these societies.

Taken together, our findings highlight the fact that more research is needed to understand the relationship between the acquisition of complex subsistence skills and unique features of human life history and cognition. For example, the effects of strength, size, practice, and their complex interactions on the development of subsistence knowledge and skill remain to be determined. Further, age of acquisition may be later, and the role of teaching may be stronger, for learning complex cultural norms and religious practices than for readily observable subsistence tasks. Beyond task difficulty, various studies suggest that birth order may impact aspects of personality and fertility across cultures (e.g., Draper and Hames 2000; Salmon and Daly 1998) and may also influence how and from whom social learning occurs. Cross-cultural studies comparing foragers with farmers can also shed light on how aspects of social structure (hierarchy vs. egalitarianism, sedentarism vs. mobility) affect how and from whom children learn. These individual- and group-level differences in learning are important avenues for future research.

## Supplementary Information

ESM 1 (PDF 2921 kb)

## Footnotes

1 The BaYaka who participated in the present study are most closely related to the Mbendjele BaYaka.
