# Supplementary material for: The Life History of Learning Subsistence Skills among Hadza and BaYaka Foragers from Tanzania and the Republic of Congo
Source: Hum Nat. 2021 May 13;32(1):16–47. doi: 10.1007/s12110-021-09386-9 (PMC8208923; doi:10.1007/s12110-021-09386-9)
Supplement: Supplementary file 1 — (PDF 2921 kb) [file 12110_2021_9386_MOESM1_ESM.pdf]

Supplementary material for: The life history of learning subsistence skills among Hadza and BaYaka foragers from Tanzania and the Republic of Congo

Authors: Sheina Lew-Levy, Erik J. Ringen, Alyssa N. Crittenden, Ibrahim A. Mabulla, Tanya Broesch, Michelle A. Kline

Table of Contents

Task Performance by Age 2
Individual Differences in Subsistence Learning 7
Subsistence Skills Free-list Nominations 8
Subsistence Task Details 9
Task Ranking Reliability 10
Task Ranking by Sex 11
Learning Method and Pathway Responses by Age 12
Additional Parameter Estimates 13
  Age-structured learning parameters 13
  Learning method parameters 14
  Transmission pathway parameters 15
Posterior correlations between task random effects 16

Task Performance by Age

Task Performance by Age: Hadza (1/2)

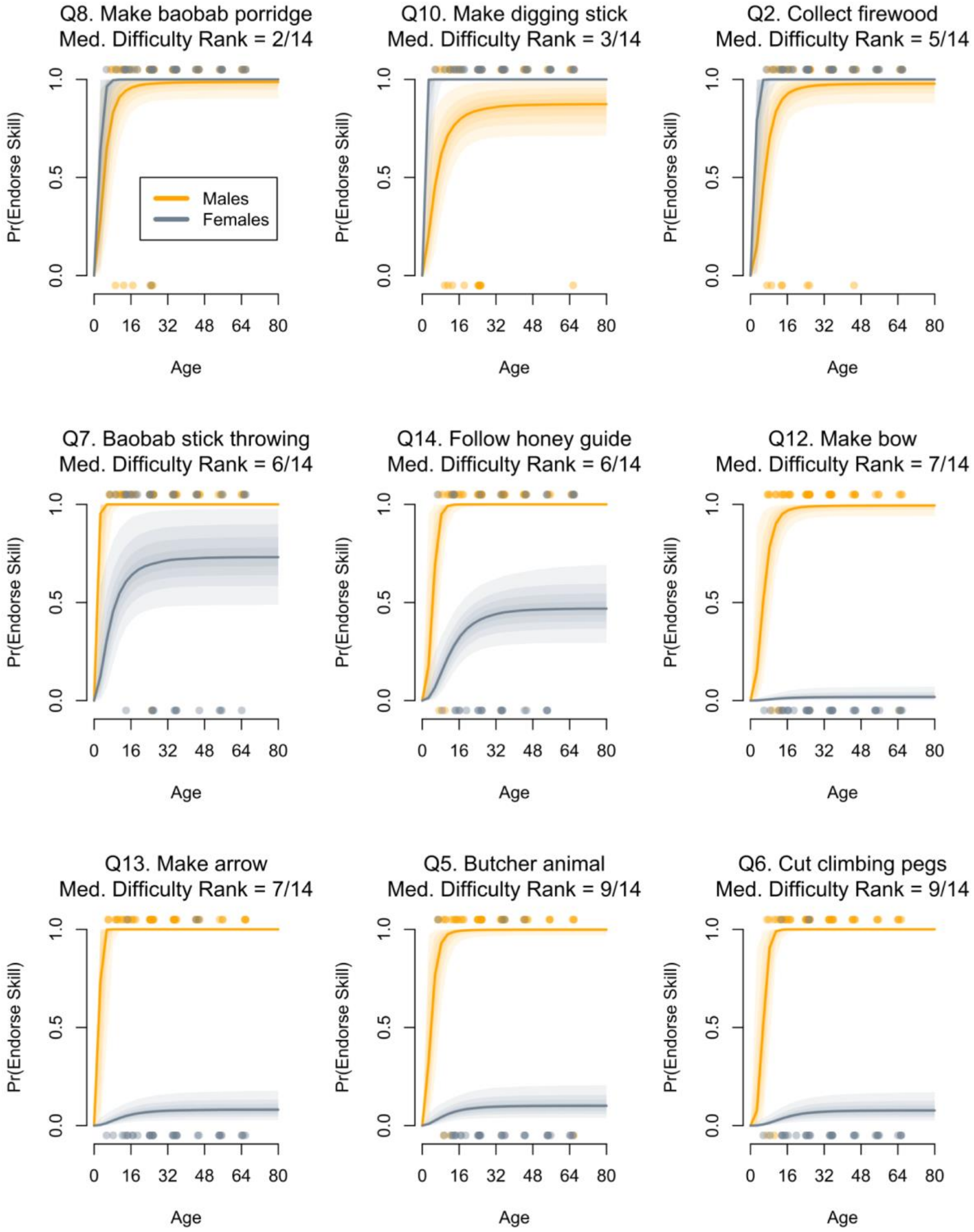

Task Performance by Age: Hadza (2/2)

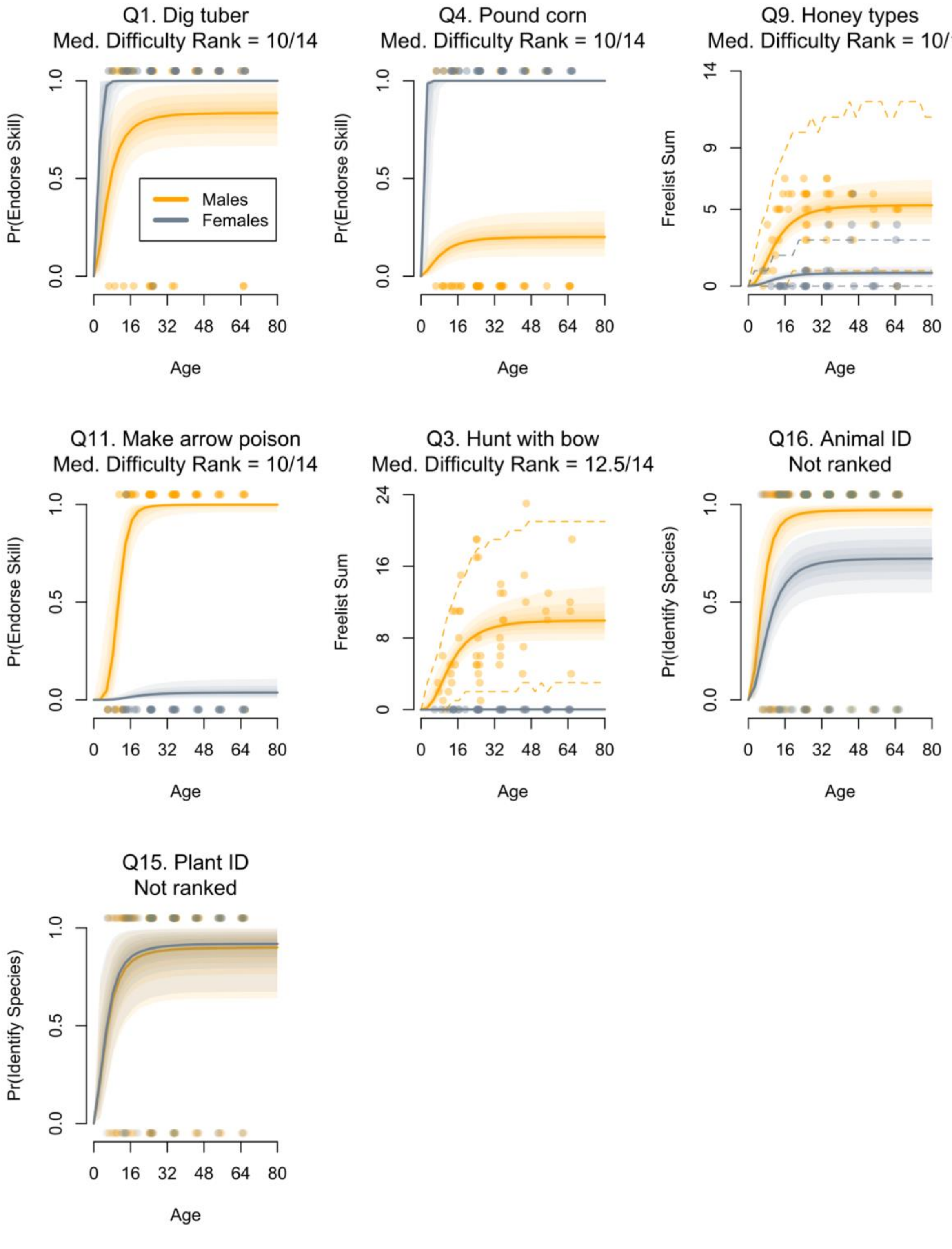

Figure S1. Counterfactual predictions of implicit and explicit subsistence responses as a function of age for the Hadza, averaging over individual differences. Males are plotted in orange and females are plotted in grey. Shaded intervals are quantiles corresponding to the 90th percentile posterior interval of the expected value for each response. Dotted intervals denote the 90th percentile of the free-list tasks, accounting for overdispersion and Poisson variance. Raw data values are plotted with a small amount of random noise to enhance visualization.

Task Performance by Age: BaYaka (1/3)

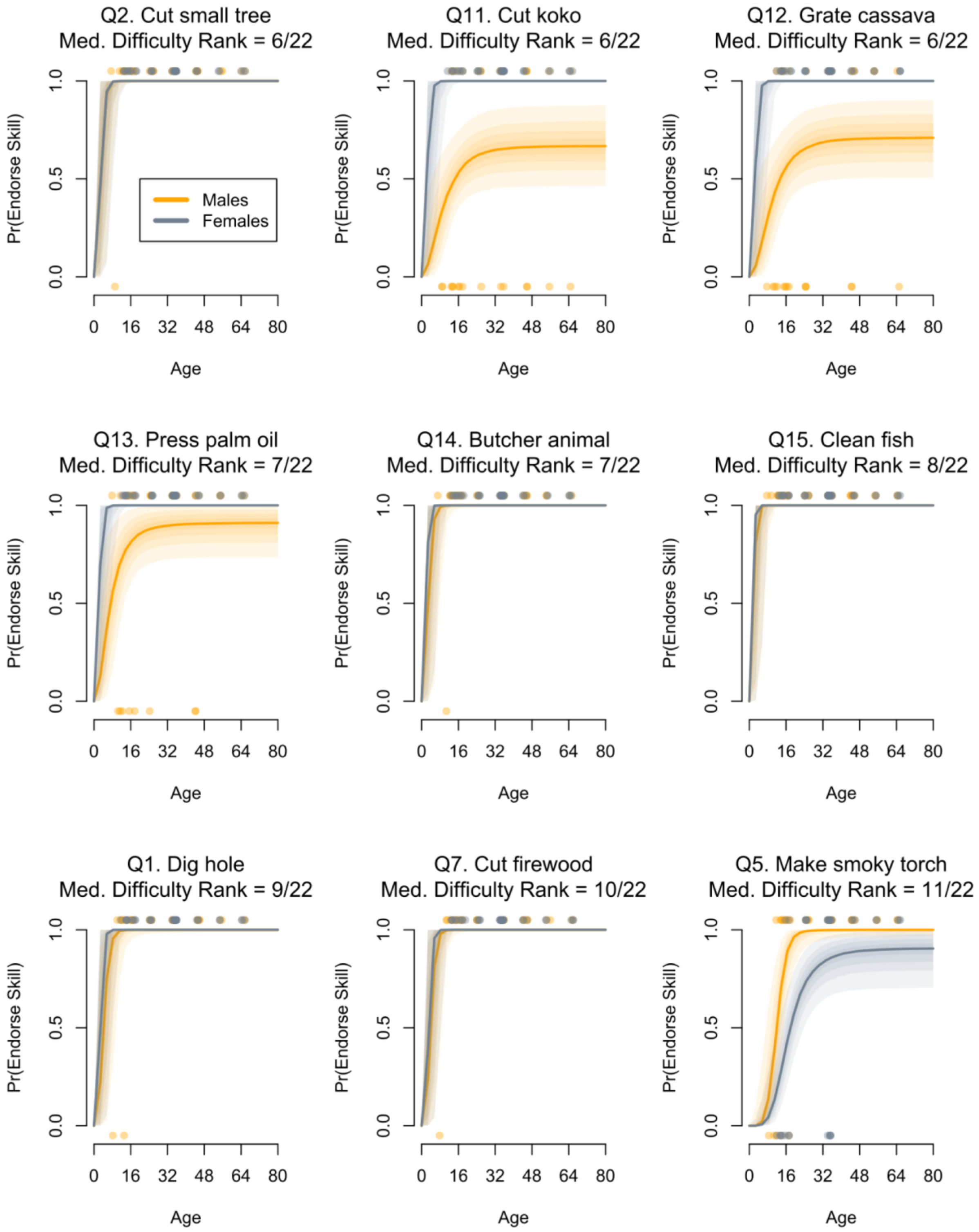

Task Performance by Age: BaYaka (2/3)

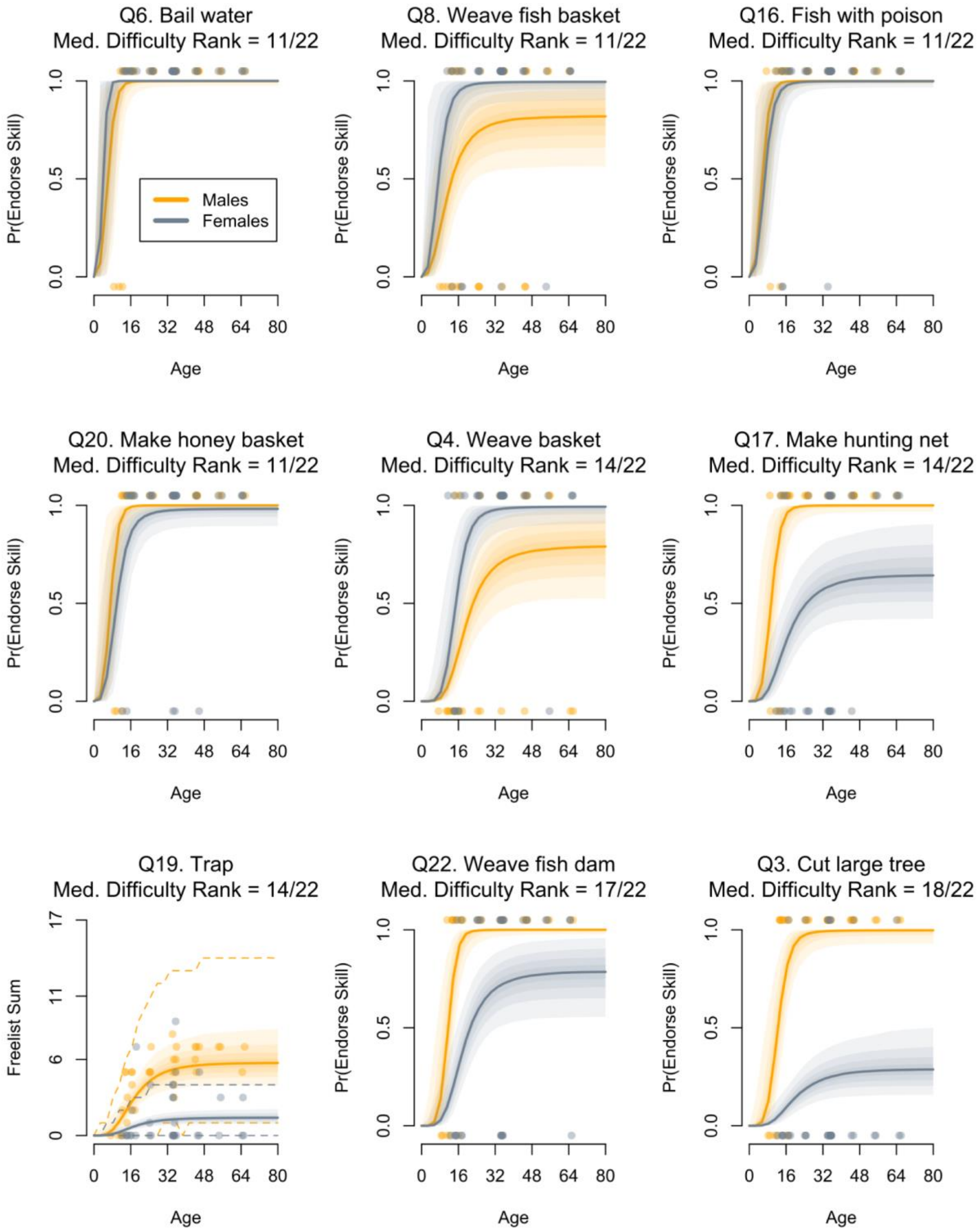

Task Performance by Age: BaYaka (3/3)

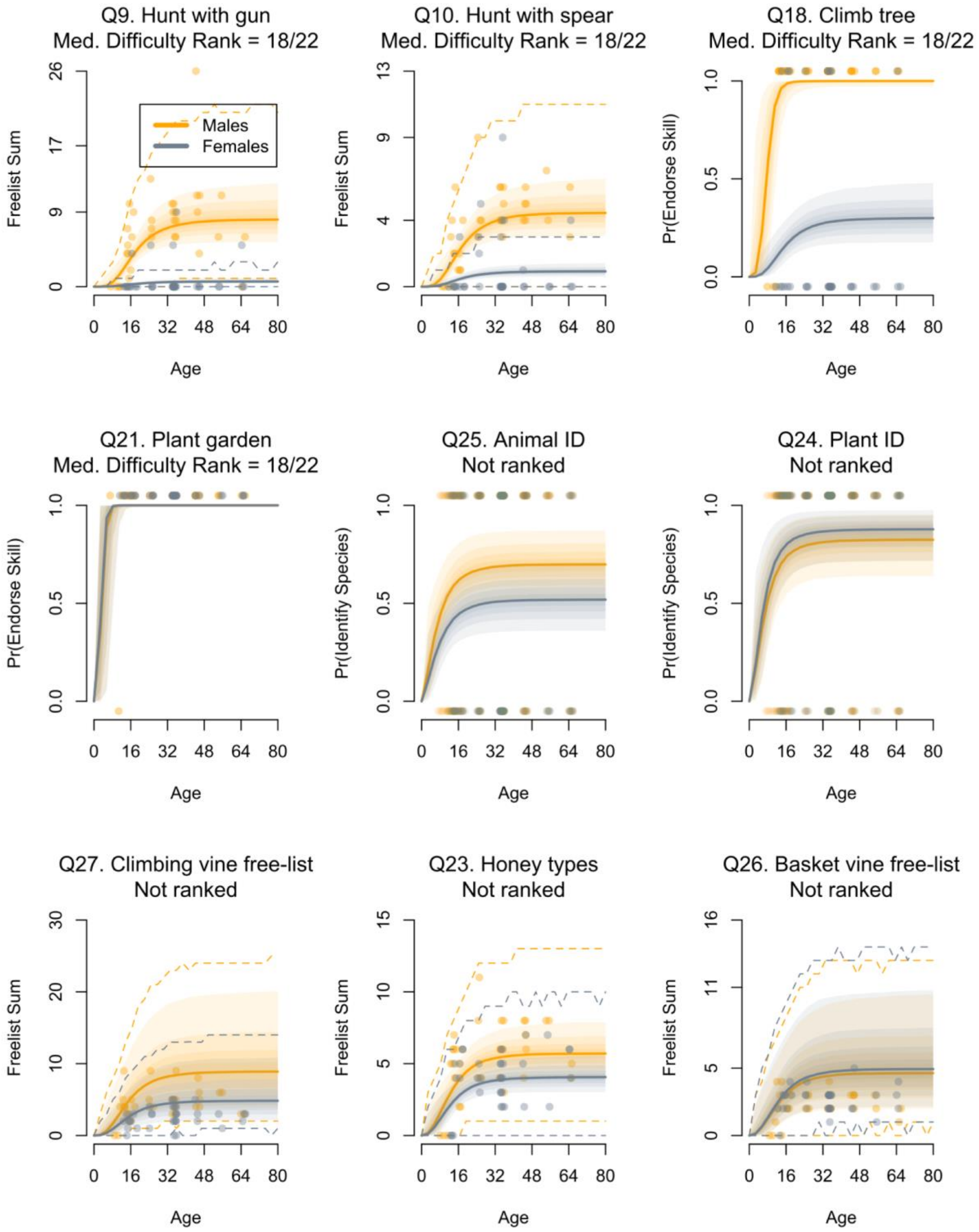

Figure S2. Counterfactual predictions of implicit and explicit subsistence responses as a function of age for the BaYaka, averaging over individual differences. Males are plotted in orange and females are plotted in grey. Shaded intervals are quantiles corresponding to the 90th percentile posterior interval of the expected value for each response. Dotted intervals denote the 90th percentile of the free-list tasks, accounting for overdispersion and Poisson variance. Raw data values are plotted with a small amount of random noise to enhance visualization.

Individual Differences in Subsistence Learning

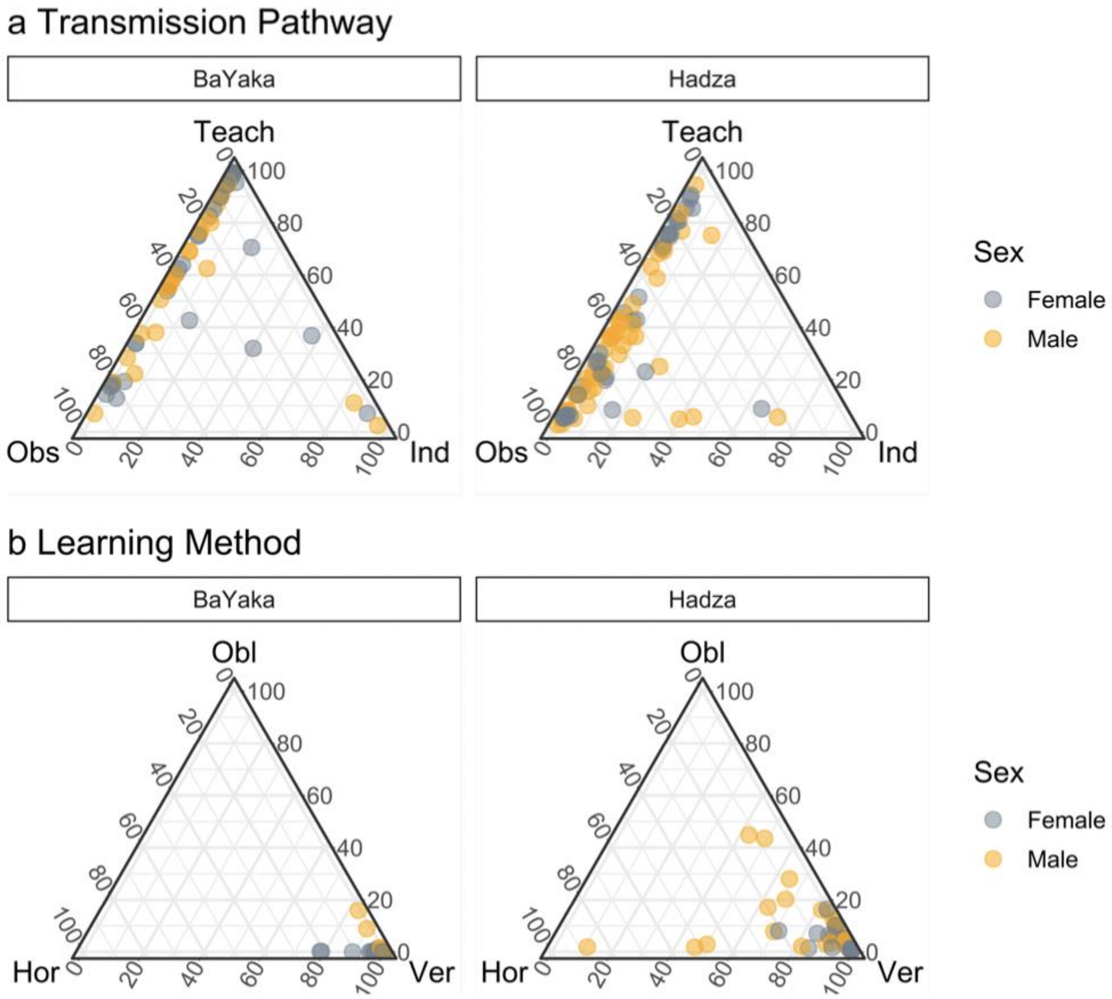

Figure S3. Ternary plots. Each point represents an individual's median posterior probability of using a given learning (a) pathway and (b) method. These coordinates can be interpreted as the reported social learning strategies of individuals. Hadza exhibit more inter-individual variation in both learning method and pathway.

Subsistence Skills Free-list Nominations

Table S1. Free-list of subsistence tasks considered important by BaYaka participants. If a participant listed two activities from the same domain (e.g. collecting *mela* tubers and collecting *ekule* tubers), these were counted as a single nomination for the 'Tubers' domain.

Domain	Task	No. nominations	No. Female Nominations
Tubers	*Mela* tubers	13	8
Tubers	*Ekule* tubers	6	4
Tubers	General tuber collecting	3	2
Hunting & trapping	Trapping	5	1
Hunting & trapping	Spear hunting	4	1
Hunting & trapping	Gun hunting	3	0
Hunting & trapping	Net hunting	9	4
Hunting & trapping	Hunting with dogs	2	0
House building	House building	2	0
Honey	Honey	7	3
General collecting	Collecting caterpillars	1	0
General collecting	Collecting *kana* nuts	1	0
General collecting	Collecting *payo* nuts	1	0
General collecting	Collecting *koko* leaves	6	5
General collecting	Collecting *pusa* seeds	2	1
General collecting	Collecting palm nuts	1	0
Garden	Gardening	6	4
Fishing	Bail fishing	3	0
Fishing	Dam fishing	1	0
Fishing	Basket fishing	1	0
Cooking	Cooking	1	1
Bowl making	Bowl making	1	0
Basketry	Basketry	4	4

Subsistence Task Details

Table S2. Tacit and explicit knowledge questionnaire administered among the Hadza and BaYaka.

**Hadza**			**BaYaka**		
Questions	Response type	Max score	Questions	Response type	Max score
Tacit Questionnaire					
Q1. Do you know how to dig up a tuber?	Binary	1	Q1. Do you know how to dig a hole?	Binary	1
Q2. Do you know how to collect firewood?	Binary	1	Q2. Do you know how to cut a small tree?	Binary	1
Q3. What types of animals have you harvested with a bow?	Free-list	23	Q3. Do you know how to cut a large tree?	Binary	1
Q4. Do you know how to pound corn?	Binary	1	Q4. Do you know how to weave a basket?	Binary	1
Q5. Do you know how to butcher animals?	Binary	1	Q5. Do you know how to make a smoky torch?	Binary	1
Q6. Do you know how to cut pegs for climbing trees?	Binary	1	Q6. Do you know how to bail water?	Binary	1
Q7. Do you know how to collect baobab via stick throwing?	Binary	1	Q7. Do you know how to cut firewood?	Binary	1
Q8. Do you know how to make baobab porridge?	Binary	1	Q8. Do you know how to weave a fish basket?	Binary	1
Q9. What types of honey have you collected?	Free-list	7	Q9. What types of animals have you harvested with a gun?	Free-list	26
Q10. Do you know how to make a digging stick?	Binary	1	Q10. What types of animals have you harvested with a spear?	Free-list	9
Q11. How do you make poison for arrows?	Binary	1	Q11. Do you know how to cut *koko* leaves?	Binary	1
Q12. Do you know how to make a bow?	Binary	1	Q12. Do you know how to grate cassava leaves?	Binary	1
Q13. How do you make an arrow?	Binary	1	Q13. Do you know how to press palm oil?	Binary	1
Q14. Do you know how to follow a honey guide?	Binary	1	Q14. Do you know how to butcher an animal?	Binary	1
			Q15. Do you know how to clean a fish?	Binary	1
			Q16. Do you know how to fish with poison?	Binary	1
			Q17. Do you know how to make a net (for hunting)?	Binary	1
			Q18. Do you know how to climb a tree with vines?	Binary	1
			Q19. What types of animals have you harvested with a trap?	Free-list	9
			Q20. Do you know how to weave a honey basket?	Binary	1
			Q21. Do you know how to plant a garden?	Binary	1
			Q22. Do you know how to weave a fishing dam?	Binary	1
			Q23. What types of honey have you collected?	Free-list	11
Explicit Questionnaire					
Q15. Plant identification	ID	8	Q24. Plant identification	ID	2016: 12<br>2017: 14
Q16. Animal identification	ID	14	Q25. Animal identification	ID	14
			Q26. Name vines used for basket weaving	Free-list	5
			Q27. Name vines used for climbing	Free-list	9

Task Ranking Reliability

We calculated Cronbach's α to assess the reliability of the task difficulty rankings. However, the usefulness of this statistic has faced stark criticism in recent years (Sijtsma 2009). Therefore, we also visualize the variation across individuals in the rank data. We present a comparison of the observed variance in ranks across individual rankers to the expected variance under a null model of random ranking (given a set number of rankers and tasks to rank). Despite high α values, it is clear that there is substantial individual variation in task difficulty rankings. Our statistical approach accounts for this heterogeneity by representing difficulty rankings as parameters, maintaining uncertainty rather than reducing them to point-estimates before model fitting.

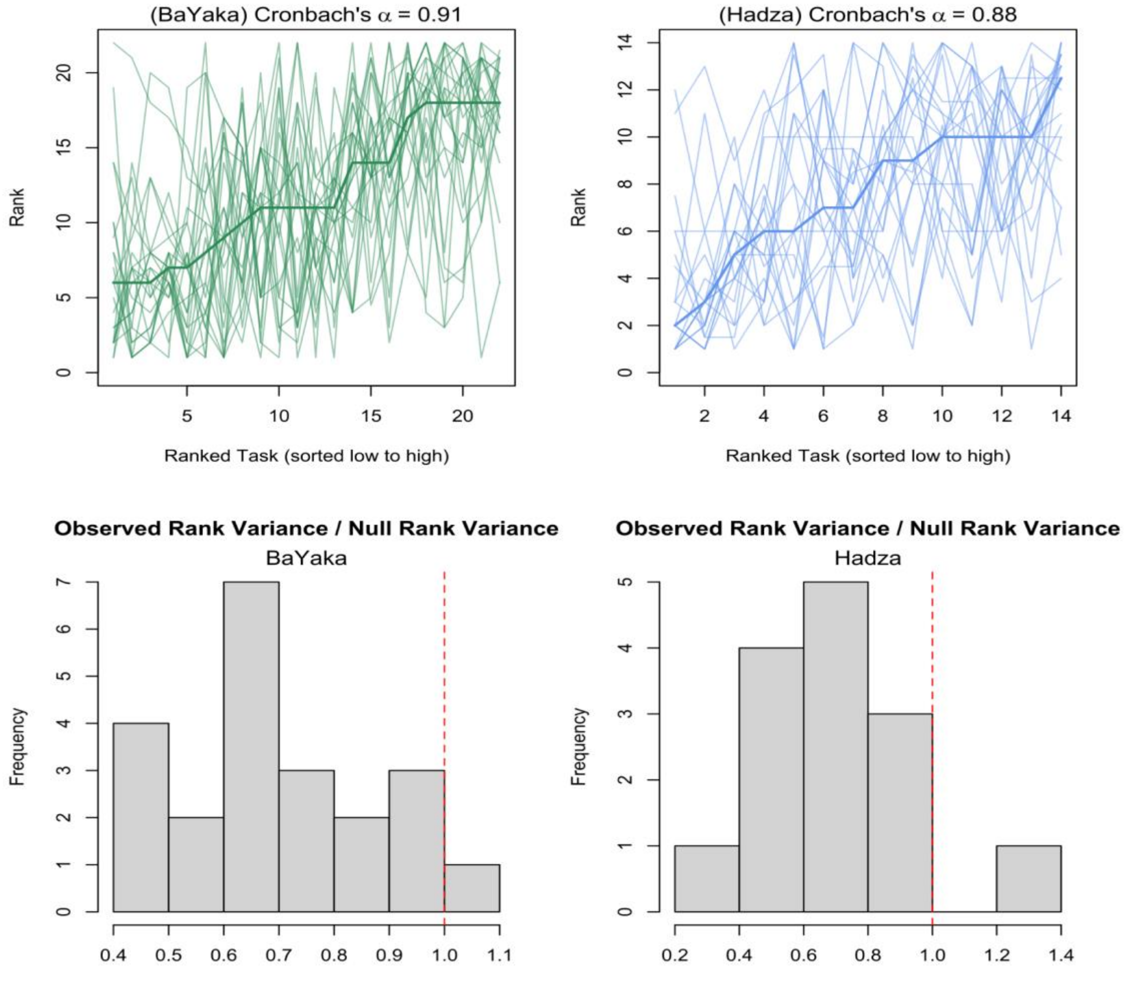

Figure S4. ***Top:*** Task difficulty rankings, with the dark line representing median ranks and each light line representing the ranks of a single participant. ***Bottom:*** Distribution of the observed within-task rank variance compared to the expected variance under a null model of random rankings. All tasks to the left of the dashed line are less variable than expected under the null distribution.

Task Ranking by Sex

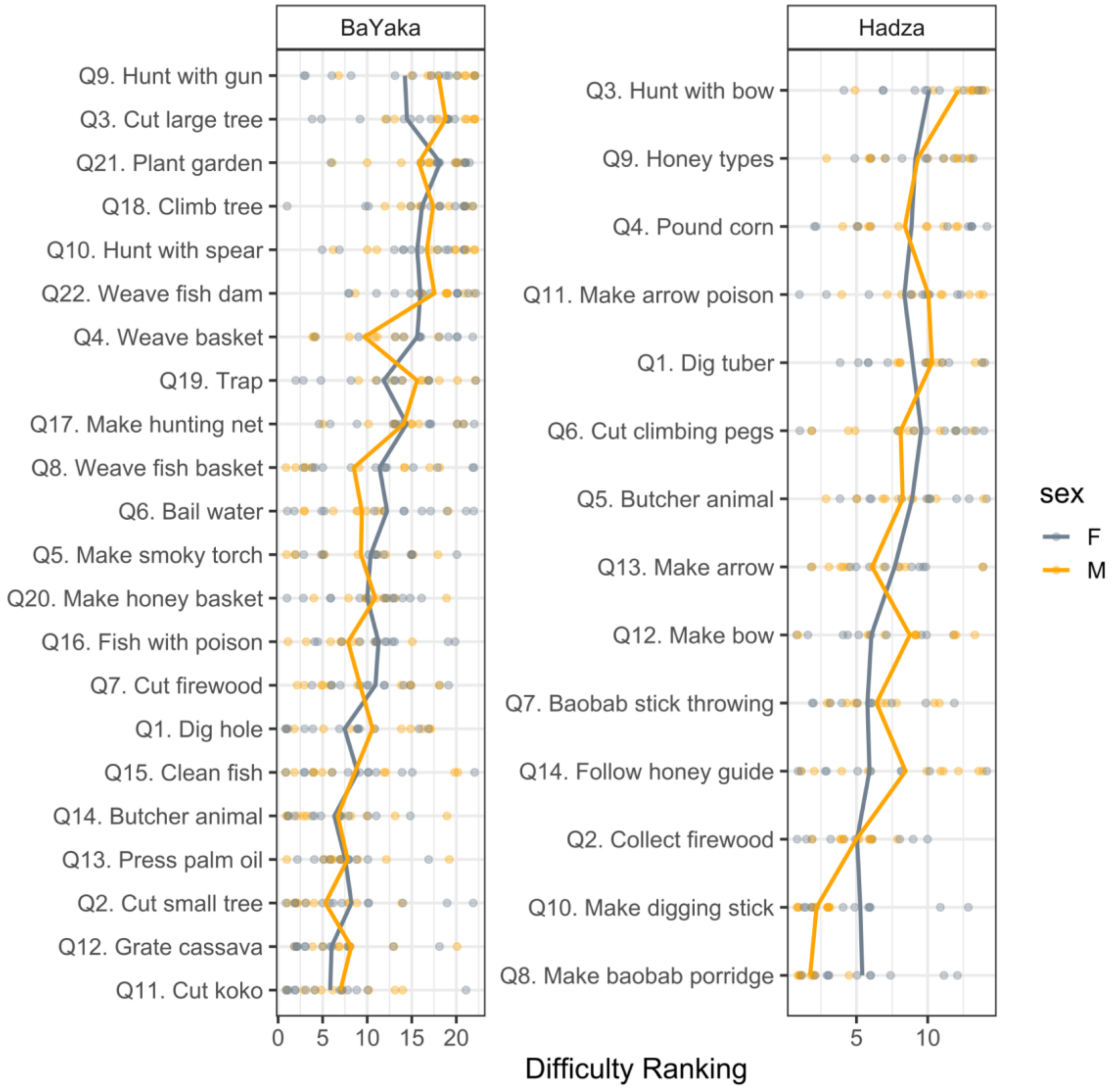

Figure S5. Task difficulty rankings sorted by median rank. Orange lines denote male median ranks, gray lines denote female median ranks. Points are individual ranks for each task, also colored according to sex.

Learning Method and Pathway Responses by Age

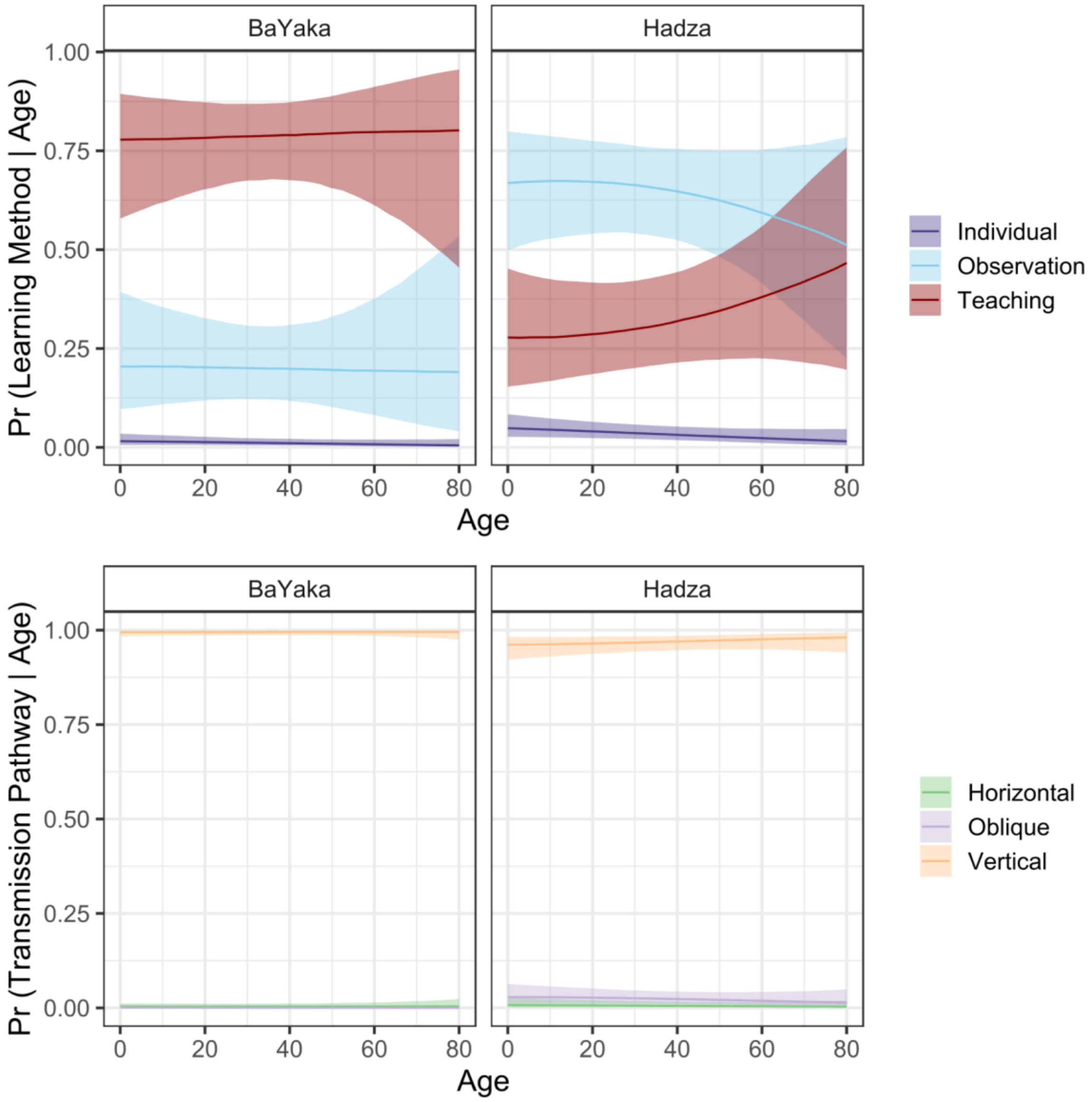

Figure S6. Posterior probabilities (median and 90% PI) for each learning method and transmission pathway as a function of age. We included second-degree polynomial terms in the models to account for potential age-related recall in learning. Except for a slight tendency for older Hadza to report teaching over observational learning, there were no strong age effects. Note that these effects cannot be interpreted as changes in learning strategies with age because participants were asked to recall how they learned a given task, which may have happened decades earlier.

Additional Parameter Estimates

Age-structured learning parameters

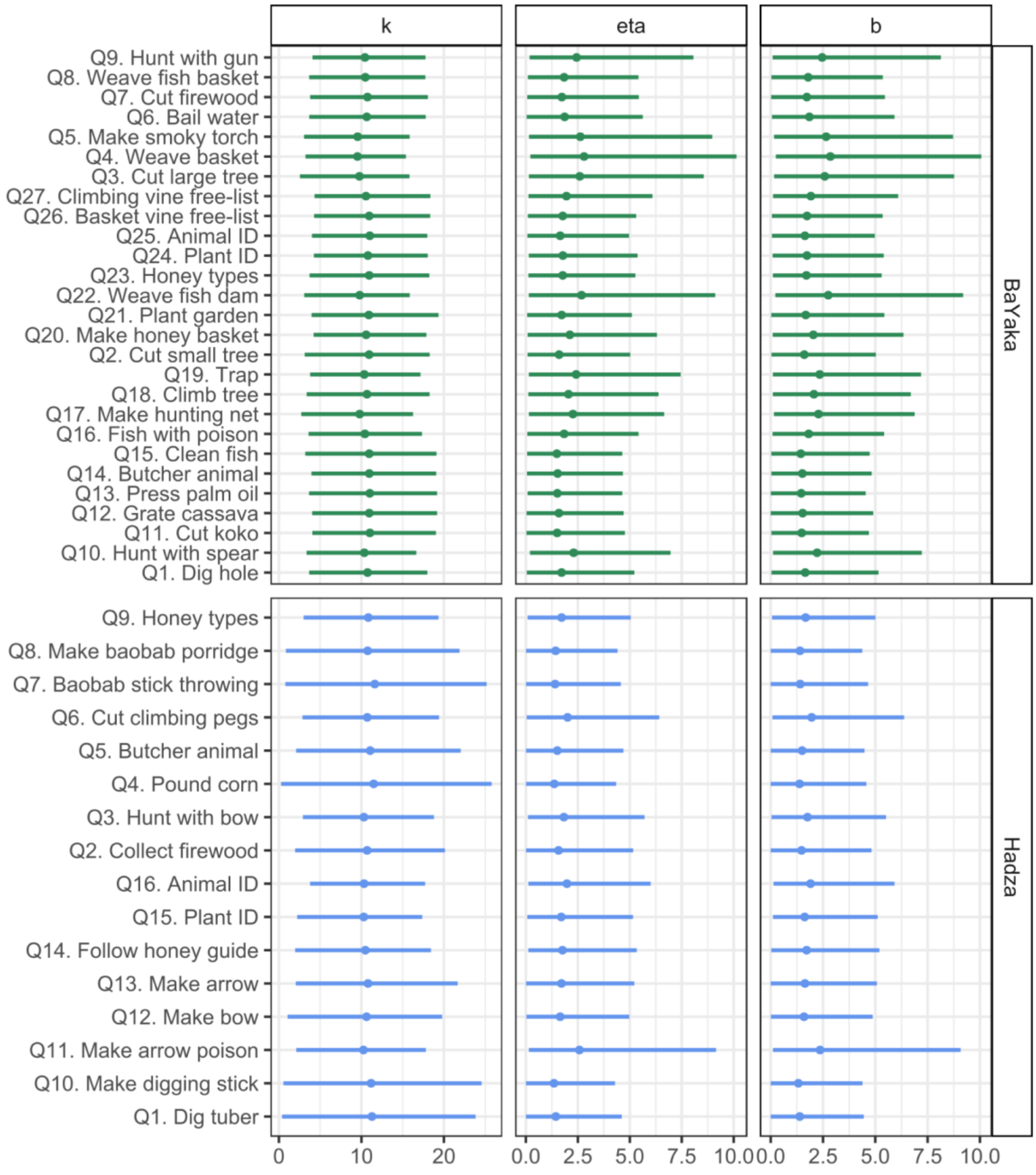

Figure S7. Posterior parameter estimates (median and 90% HPDI) for each component of the age-structured learning model, broken up by subsistence task.

Learning method parameters

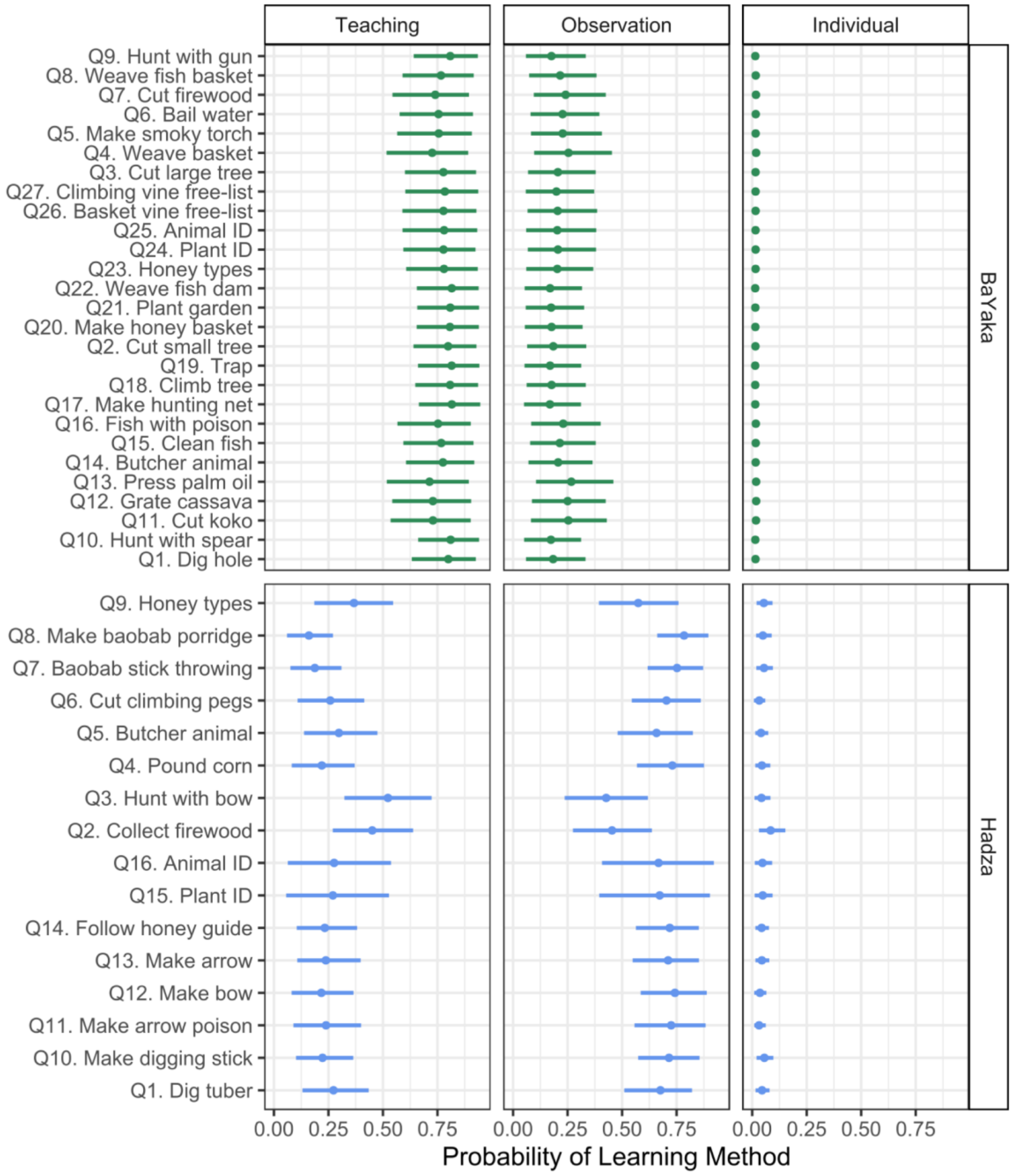

Figure S8. Posterior parameter estimates (median and 90% HPDI) for the probability of each learning method, broken up by subsistence task.

Transmission pathway parameters

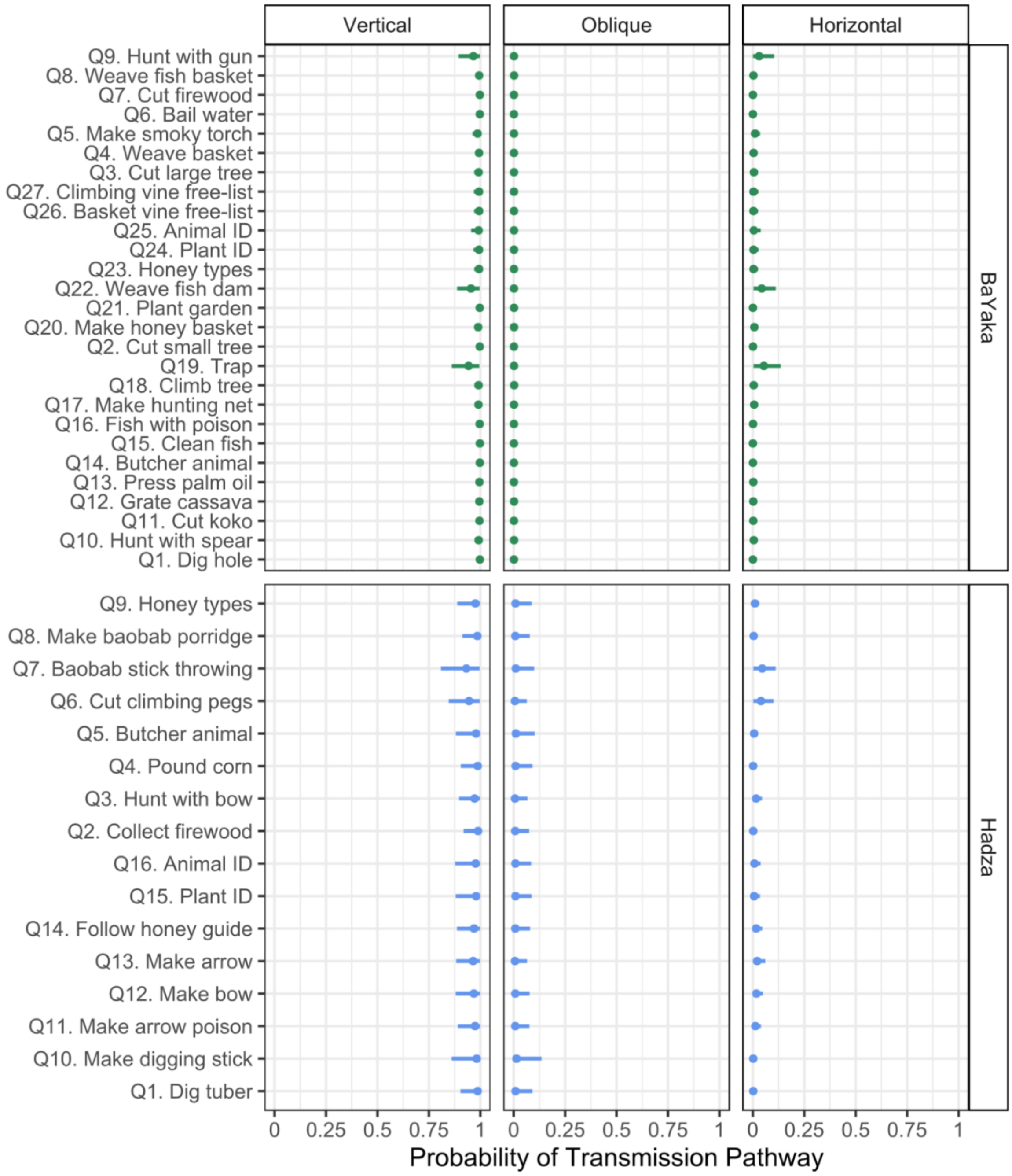

Figure S9. Posterior parameter estimates (median and 90% HPDI) for the probability of each transmission pathway, broken up by subsistence task.

Posterior correlations between task random effects

Table S3. Posterior median task-level correlations between model components (BaYaka).

	k	*b*	*eta*	*a*	*a_male*	*horiz*	*obl*	*obs*	*teach*	*female_transmission*	*rank*
k		0.03	0.02	0.03	-0.08	-0.06	0.01	-0.01	-0.00	0.07	-0.06
b	0.03		-0.03	-0.11	0.16	0.12	0.00	0.00	0.01	-0.14	0.14
eta	0.02	-0.03		-0.11	0.15	0.12	0.01	0.01	0.01	-0.14	0.14
a	0.03	-0.11	-0.11		-0.51	-0.33	-0.13	0.02	-0.07	0.56	-0.37
a_male	-0.08	0.16	0.15	-0.51		0.15	0.05	-0.09	0.21	-0.60	0.39
horiz	-0.06	0.12	0.12	-0.33	0.15		0.05	-0.02	0.06	-0.35	0.13
obl	0.01	0.00	0.01	-0.13	0.05	0.05		-0.02	0.04	-0.07	0.11
obs	-0.01	0.00	0.01	0.02	-0.09	-0.02	-0.02		0.02	0.08	-0.08
teach	-0.00	0.01	0.01	-0.07	0.21	0.06	0.04	0.02		-0.20	0.15
female_transmission	0.07	-0.14	-0.14	0.56	-0.60	-0.35	-0.07	0.08	-0.20		-0.33
rank	-0.06	0.14	0.14	-0.37	0.39	0.13	0.11	-0.08	0.15	-0.33	

Table S4. Posterior median task-level correlations between model components (Hadza).

	k	*b*	*eta*	*a*	*a_male*	*horiz*	*obl*	*obs*	*teach*	*female_transmission*	*rank*
k		0.04	0.04	0.01	-0.04	-0.01	0.03	0.01	-0.03	0.03	-0.01
b	0.04		-0.02	-0.06	0.07	0.04	-0.04	0.02	0.05	-0.08	0.05
eta	0.04	-0.02		-0.06	0.07	0.05	-0.04	0.01	0.05	-0.08	0.05
a	0.01	-0.06	-0.06		-0.65	-0.23	0.09	-0.15	-0.19	0.47	-0.25
a_male	-0.04	0.07	0.07	-0.65		0.35	-0.09	0.09	0.14	-0.54	0.13
horiz	-0.01	0.04	0.05	-0.23	0.35		-0.03	0.10	-0.02	-0.32	0.02
obl	0.03	-0.04	-0.04	0.09	-0.09	-0.03		0.01	-0.05	0.08	-0.05
obs	0.01	0.02	0.01	-0.15	0.09	0.10	0.01		0.08	-0.02	0.02
teach	-0.03	0.05	0.05	-0.19	0.14	-0.02	-0.05	0.08		-0.12	0.26
female_transmission	0.03	-0.08	-0.08	0.47	-0.54	-0.32	0.08	-0.02	-0.12		-0.11
rank	-0.01	0.05	0.05	-0.25	0.13	0.02	-0.05	0.02	0.26	-0.11	

References

Sijtsma, K. (2009). On the use, the misuse, and the very limited usefulness of cronbach's alpha. *Psychometrika*, *74*(1), 107–120. doi:10.1007/s11336-008-9101-0
